# Combining thermal scanning probe lithography and dry etching for grayscale nanopattern amplification

**DOI:** 10.1038/s41378-024-00655-y

**Published:** 2024-02-23

**Authors:** Berke Erbas, Ana Conde-Rubio, Xia Liu, Joffrey Pernollet, Zhenyu Wang, Arnaud Bertsch, Marcos Penedo, Georg Fantner, Mitali Banerjee, Andras Kis, Giovanni Boero, Juergen Brugger

**Affiliations:** 1 Microsystems Laboratory, École Polytechnique Fédérale de Lausanne (EPFL), Lausanne, 1015 Switzerland; 2 Center of MicroNanoTechnology (CMi), EPFL, Lausanne, 1015 Switzerland; 3 Laboratory of Nanoscale Electronics and Structures, École Polytechnique Fédérale de Lausanne (EPFL), Lausanne, 1015 Switzerland; 4 Laboratory for Bio- and Nano- Instrumentation, École Polytechnique Fédérale de Lausanne (EPFL), Lausanne, 1015 Switzerland; 5 Laboratory of Quantum Physics, Topology and Correlations, École Polytechnique Fédérale de Lausanne (EPFL), Lausanne, 1015 Switzerland; 6 Present Address: Institute of Materials Science of Barcelona ICMAB-CSIC, Campus UAB, Bellaterra, 08193 Spain; 7 Present Address: School of Integrated Circuits and Electronics, MIIT Key Laboratory for Low-Dimensional Quantum Structure and Devices, Beijing Institute of Technology, Beijing, 100081 China

**Keywords:** Nanoscale devices, Nanoscale materials, Nanoscale materials

## Abstract

Grayscale structured surfaces with nanometer-scale features are used in a growing number of applications in optics and fluidics. Thermal scanning probe lithography achieves a lateral resolution below 10 nm and a vertical resolution below 1 nm, but its maximum depth in polymers is limited. Here, we present an innovative combination of nanowriting in thermal resist and plasma dry etching with substrate cooling, which achieves up to 10-fold amplification of polymer nanopatterns into SiO_2_ without proportionally increasing surface roughness. Sinusoidal nanopatterns in SiO_2_ with 400 nm pitch and 150 nm depth are fabricated free of shape distortion after dry etching. To exemplify the possible applications of the proposed method, grayscale dielectric nanostructures are used for scalable manufacturing through nanoimprint lithography and for strain nanoengineering of 2D materials. Such a method for aspect ratio amplification and smooth grayscale nanopatterning has the potential to find application in the fabrication of photonic and nanoelectronic devices.

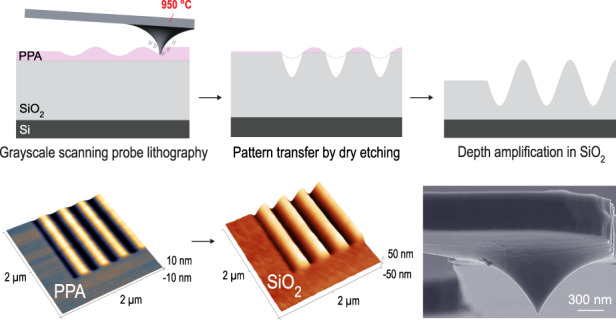

## Introduction

Nanotechnologies have been consistently advancing with the improvement of lithography techniques^1,2^. Grayscale lithography at the sub-micrometer scale has gained attention for its ability to create 2.5D topographies with multiple depth levels, leading to enhanced functionality of micro- and nanodevices such as diffractive optical elements^3^ and microelectromechanical systems^4^. Grayscale electron beam lithography (EBL) has been used to fabricate staircase patterns with a step height of 6 nm and width down to 32 nm^5^. The lateral and vertical resolution of this lithography process is limited by electron beam scattering. Interference lithography is another way to create surface topographies with varying height levels. The vertical and lateral resolution of the resulting grayscale nanopatterns is constrained by the exposure wavelength, as well as the intensity and phase of the interfering light waves^6^.

Thermal scanning probe lithography (t-SPL) has also been used for grayscale nanopatterning, demonstrating lateral spatial resolutions down to the single-digit nanometer and sub-nanometer vertical depth control^7–10^. The thermal spreading effect caused by the heated tip in t-SPL is not as severe as the proximity effect caused by electron scattering in EBL. In particular, the endothermic decomposition reaction of the thermally-sensitive resist, polyphthalaldehyde (PPA), localizes patterning at the contact area of the tip, enabling sub-10 nm lateral resolutions with sharp tips. PPA sublimates within microseconds^11,12^, allowing for rapid patterning at scan speeds of around 1 mm/s, which are comparable to EBL^9^. t-SPL speeds up to 20 mm/s have also been reported^13^. The combined write-and-read mechanism of t-SPL, called closed-loop lithography, enables simultaneous correction of the patterned depths, achieving sub-nanometer vertical depth control^14^.

t-SPL has been used for both high-resolution binary^15–21^ and grayscale patterning^14,22–26^. Single-digit nanometer resolution grayscale lithography with sub-1 nm vertical depth control has been successfully implemented in a nanofluidic device that can separate nanoparticles with a diameter variation of only 1 nm^22^ and has also been used for the fabrication of sinusoidal gratings for more precise control over a diffracted wavefront compared to binary-shaped conventional gratings^25^. The grayscale fabrication capability of t-SPL enables the fabrication of sinusoidal patterns with adaptable amplitude, spatial frequency, and phase^25^. Despite the impressive nanofabrication results achieved using t-SPL, one of the main challenges lies in achieving large patterning depths. The practical nanostructure patterning depth limit in PPA with t-SPL is about 100 nm due to the probe and tip geometry as well as the thermomechanical response of the polymer resist^17^. Deeper writing in the resist reduces heating efficiency due to tip cooling, particularly for sharper tips where the boundary scattering of phonons further decreases the efficiency^27,28^. This shallow patterning depth limits the use of t-SPL for applications requiring high aspect ratio grayscale nanopatterning, such as optical diffractive elements^29,30^. In addition, the depth of sub-10 nm patterns is typically limited to values below 10 nm due to the conical geometry of the tip^31,32^. To achieve larger depths, pattern transfer from PPA into multiple underlying layers is used. However, this approach has been demonstrated only for binary patterns, and each pattern transfer process caused feature widening, in turn affecting the achievable lateral resolution^31,32^. Non-binary transfer has been shown for saw-tooth and sinusoidal profile transfer from PPA to SiN_x_, but with significant shape deformation and large depth amplification variation^23^. As an alternative approach, cyclic infiltration of inorganic materials has been shown to convert patterned PPA resists into more etch-resistant inorganic hard masks, allowing for the amplification of depths in underlying layers after pattern transfer^33^. However, this approach requires additional chemical post-processing steps on PPA.

To overcome these limitations in patterning depth, in this work, we demonstrate an advanced combination of grayscale patterning in PPA and dry etching-based transfer into dielectric layers to amplify the depths of grayscale nanopatterns through optimized etching parameters with cooling cycles. The reported etch transfer and pattern amplification approach is generic and applicable to various polymer/dielectric stacks, regardless of the lithography techniques employed. To exemplify the possible applications of the proposed approach, we use grayscale dielectric nanostructures as thermal nanoimprint lithography (NIL) stamps for scalable replication and as a platform to induce local strain in atomically thin MoS_2_ layers.

## Results and Discussion

### Grayscale nanopatterning and depth amplification

**Fig. 1:**
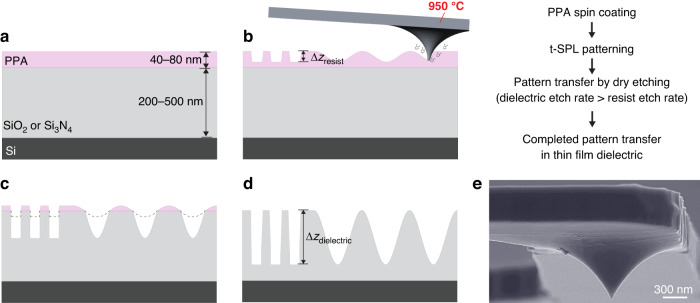
Combination of grayscale t-SPL and dry etching. Cross-sectional illustration of the process flow for grayscale nanopattern amplification in dielectrics. **a** Spin-coating of the thermally-sensitive resist PPA on a thin dielectric film, in our case SiO_2_ or Si_3_N_4_. **b** Fabrication of binary and grayscale nanostructures on a thin layer of PPA using a heated nanotip (see Figure S2 for the details of the nanotip). **c** Transfer of the nanostructures from PPA to SiO_2_ or Si_3_N_4_. **d** Complete transfer of the nanostructures written in PPA into the dielectric film with depth amplification. The vertical peak-to-peak depth amplification (Δ*z*_dielectric_/Δ*z*_resist_) results from the difference in etch rates between the resist and substrate in CHF_3_/SF_6_ plasma. Illustration images are not to scale. **e** SEM image of the tip of the cantilever (NanoFrazor Monopede, Heidelberg Instruments Nano AG)

In the following, we describe the process for grayscale nanopattern amplification based on the combination of t-SPL and dry etching. As illustrated in Fig. 1, a silicon substrate with a thin film of either SiO_2_ or Si_3_N_4_ is spin-coated with thermally-sensitive PPA resist and grayscale nanopatterns are fabricated by direct sublimation of the resist according to the pre-defined depths for each pixel of the grayscale bitmap (see Figure S1 for bitmap design). Binary and sinusoidal patterns are fabricated with sub-nanometer depth control using t-SPL. The sinusoidal nanopatterns with a pitch of 400 nm and peak-to-peak depths of up to 30 nm are defined by the equation $f(x,y)=A\cos (gx)$, where *A* and *g* are amplitude and spatial frequency, respectively. Patterning at peak-to-peak depths larger than 30 nm significantly reduces the lateral resolution due to the tip’s conical geometry (Figures 1 and S2). Deeper tip indentations result in broader and non-symmetrical patterns, as we experimentally show in Figure S3. In addition to the tip apex, the slope of the tip also plays a role in heat transfer for deep indentations, preventing the fabrication of closely spaced patterns and limiting the aspect ratios of fabricated nanostructures (see Figures S4–S6). In addition to our experimental demonstrations, the minimum achievable pitch with respect to tip diameter and indentation depths has been theoretically calculated in a recent study^34^. Therefore, we limit grayscale nanopattern depths to achieve the highest spatial resolutions in sinusoidal wave patterns with pitches below 400 nm.

Following grayscale t-SPL, the nanopatterns are transferred from the resist into a dielectric layer, either SiO_2_ or Si_3_N_4_, using inductively coupled plasma (ICP) reactive ion etching (RIE). The progressive erosion of the grayscale patterns locally modifies the opening in the resist masking layer (Fig. 1c). Hence, patterns on the resist are replicated on the substrate with a depth amplification that depends on the etch selectivities of SiO_2_ to PPA or Si_3_N_4_ to PPA in CHF_3_/SF_6_ plasma. See Methods and Table S1 in the Supplementary Information for further details.

**Fig. 2:**
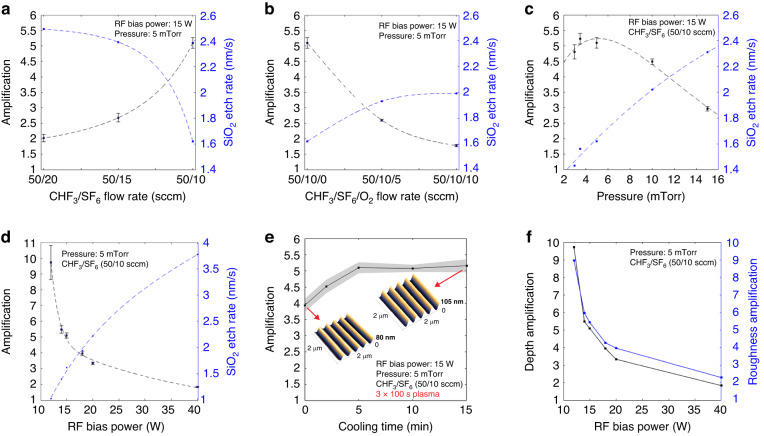
Effect of the dry etching parameters on the grayscale depth amplification. **a** Effect of CHF_3_/SF_6_ gas flow rate and **b** effect of adding O_2_ on the depth amplification and SiO_2_ etch rate at a fixed pressure of 5 mTorr and an RF bias power of 15 W. **c** Influence of chamber pressure on depth amplification and SiO_2_ etch rate for a CHF_3_/SF_6_ plasma with flow rate of 50/10 sccm at 15 W fixed RF bias power. **d** Effect of RF bias power on depth amplification for a CHF_3_/SF_6_ plasma with flow rate of 50/10 sccm at 5 mTorr fixed pressure. The error bars correspond to one standard deviation (±*σ*) for 16 to 48 surface profile comparisons, and the dashed lines represent polynomial fits of the experimental data. **e** Effect of the substrate temperature on the grayscale depth amplification. Depth amplification is obtained after three consecutive 100 s plasma etching cycles separated by substrate cooling periods of 0, 2, 5, 10, and 15 min. The shaded area represents one standard deviation (±*σ*) for 16 surface profile comparisons. In all cases (from **a** to **e**), RF ICP power and substrate cooling temperature are kept constant at 950 W and 10 °C, respectively. **f** Depth amplification and corresponding surface roughness amplification after dry etching for varying values of RF bias power

During the aspect ratio amplification process by RIE, the substrate cooling temperature was fixed to 10 °C using a temperature-controlled electrostatic clamping chuck and backside He cooling as the substrate temperature is one of the parameters that allows the control of the etch selectivities^4^. Cooling cycles of 5 min were added after every 100 s of plasma etching to prevent substrate overheating. By changing the CHF_3_/SF_6_ rate from 50/20 sccm to 50/10 sccm (at 5 mTorr and 15 W RF bias power), the etch rate of SiO_2_ decreases by 35%. However, the SiO_2_ to PPA etch selectivity, which provides the depth amplification, increases by 125% as shown in Fig. 2a. At the optimal flow rate, a depth amplification of 5 times is achieved. Further reduction of the CHF_3_/SF_6_ relative gas flow rates (50/5 sccm) induces polymerization due to CH_*x*_ and CF_*x*_ adsorption on the nanostructured surface, preventing nanopattern transfer. Adding 5 or 10 sccm O_2_ to the 50/10 sccm of CHF_3_/SF_6_ plasma results in a 49% and 65% reduction in average amplification, respectively (Fig. 2b). Higher chamber pressure results in faster SiO_2_ etching due to increased concentrations in reactive gases, but this reduces the amplification (Fig. 2c). The highest depth amplifications are observed at a pressure of 3.5 mTorr, while a reduction to 3 mTorr results in an 8% drop in average grayscale depth amplification (see Figure S7 for the comparison of amplification at 3.5 and 5 mTorr pressures). At lower pressures, physical etching dominates, leading to a reduction in etch selectivity.

The RF bias power applied to the substrate through the bottom electrode generates a self-bias that controls the reactive species bombardment energy and physical etching yield, affecting the depth amplification. Figure 2d shows the significant influence of the RF bias power on the grayscale depth amplification. An amplification of up to 10 ± 1 is achieved at 12 W, showing a 2-fold increase compared to etching at 15 W. Lower RF bias powers result in higher amplification. A further reduction of the bias power to 10 W causes polymerization, blocking pattern transfer into SiO_2_. On the contrary, higher RF bias powers lead to lower amplifications (1.2-fold at 80 W). Furthermore, CHF_3_/SF_6_ plasma is used to transfer grayscale PPA nanopatterns to Si_3_N_4_, another commonly used dielectric in semiconductor devices, but with reduced amplifications compared to SiO_2_ (see Figure S8).

Despite the continuous backside cooling, heat inflow from the plasma causes the substrate temperature to increase, reducing the sticking probability of reaction byproducts to the nanopattern surfaces. Therefore, at higher substrate temperatures, the presence of CH_*x*_ and CF_*x*_ is less efficient in protecting the sidewalls of patterns. This affects the anisotropy of the etching process^35^ and causes a reduction in the grayscale depth amplification. To prevent substrate overheating, we performed a cycled RIE process that alternates between plasma etching and cooling phases. When the dry etching process is paused for more than 5 min every 100 s of plasma etching to let the substrate cool down, the average depth amplification is increased by up to 33% compared to continuous plasma etching (Fig. 2e). Higher substrate temperatures affect the sidewall protection, in turn, reducing the depth amplification. See Figure S9 for details on the substrate cooling.

Despite the sub-nanometer depth control, the nanopatterns produced by t-SPL have a certain surface roughness^14^, which is typically about 0.4 to 0.5 nm_rms_ on the patterned PPA resist. This inherent surface roughness is typically further enhanced during the dry etch-based transfer. Substrate temperature, pressure, bias power, and the C/F ratios significantly affect the emission of fluorocarbon and silicon oxide species from the substrates and their redeposition rates^35–38^. Additionally, ion damage on the resist surface modifies the resist surface roughness^39^, which is translated to the underlying layer on the substrate. This transferred roughness can be smoothened, up to a certain limit, with post-treatments such as ion-beam milling^40^, but it is mostly efficient for flat surfaces rather than high aspect ratio nanopatterns. Therefore, minimizing the surface roughness generated by the plasma etching step while amplifying the depths of nanopatterns is crucial in achieving smooth grayscale nanostructures.

The highest amplification value reported so far in literature between PPA and SiO_2_, which is a 3-fold amplification in depth profiles of binary patterns, was achieved using C_4_F_8_/H_2_/He plasma^14^. However, the plasma used for this depth amplification process also increases the surface roughness up to 8 times, which in turn reduces the functional quality of the nanostructures for device integration. Figure 2f shows that our newly developed dry etching recipe, the CHF_3_/SF_6_ plasma (50/10 sccm) at 5 mTorr pressure with controlled substrate temperature, enables the transfer without introducing additional surface roughness. To evaluate the quality of pattern transfer in terms of roughness, we compare the ratio of depth amplification to roughness amplification. We observe that the CHF_3_/SF_6_ gas flow rate ratios have a larger effect on surface roughness than the RF bias power and pressure. For example, while the ratio is 0.94 for CHF_3_/SF_6_ plasma with a flow rate of 50/10 sccm, it is reduced to 0.41 when the SF_6_ flow rate is increased to 20 sccm under the same plasma conditions. The surface roughness is quantitatively characterized using atomic force microscopy (AFM) on both flat and sinusoidal surfaces, yielding similar values, on 2 *μ*m^2^ and 8 *μ*m^2^ areas, respectively. See Figure S10 for the comparison of our recipes with current state-of-the-art processes.

**Fig. 3:**
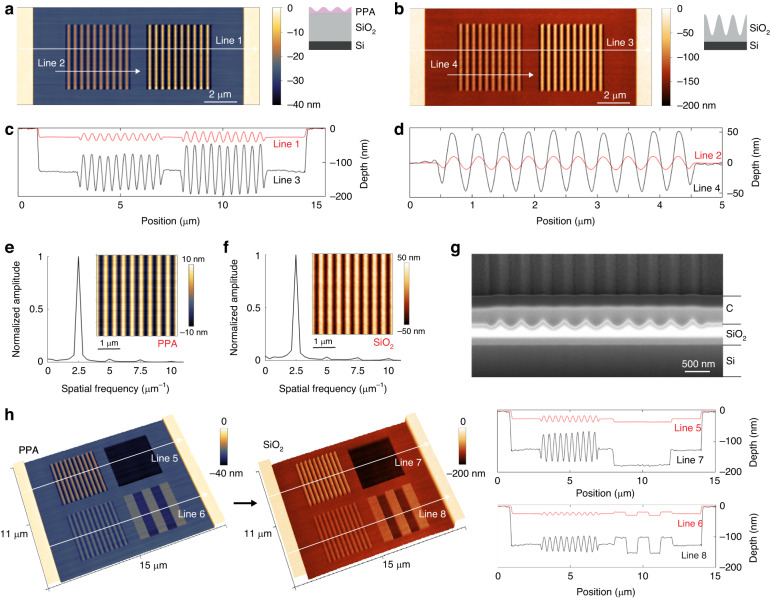
Topography characterization. **a** AFM image of sinusoidal patterns fabricated on PPA. **b** AFM image of the sinusoidal patterns shown in a after their transfer into SiO_2_ by dry etching (CHF_3_/SF_6_ plasma with flow rate of 50/10 sccm at 5 mTorr pressure, and 15 W RF bias power). **c** Comparison of the PPA and SiO_2_ cross-section profiles measured by AFM along Line 1 and Line 3. After dry etching, the sinusoidal pattern has been amplified 5 times and the sample surface has been shifted 100 nm lower in SiO_2_. **d** Comparison of the sinusoidal profiles before (Line 2) and after (Line 4) pattern transfer by dry etching. The sinusoidal pattern has been amplified in peak-to-peak amplitude from 20 nm to 100 nm. **e**, **f** Fourier transforms of the measured topographies after t-SPL on PPA (left sinusoidal nanopattern in **a**) and transferred into SiO_2_ (left sinusoidal nanopattern in **b**). **g** Tilt (54°) corrected cross-sectional SEM image of sinusoidal nanostructure fabricated on SiO_2_ by depth amplification corresponding to Line 4. The areas of interest are protected by a deposited carbon layer and milled by FIB (see Figure S15 for SEM images before and after FIB milling). **h** AFM images and surface profile comparison of rectangular and sinusoidal nanopatterns having varying aspect ratios after t-SPL patterning in PPA and pattern transfer into SiO_2_

Next, we investigate the surface topographies of PPA and dielectric layers. Figure 3 shows 5-fold amplification in depth profiles of rectangular and sinusoidal nanostructures (400 nm pitch with 10, 20 and 30 nm depths) when transferred from PPA to SiO_2_ using CHF_3_/SF_6_ plasma with a flow rate of 50/10 sccm at 950 W RF ICP power, 15 W RF bias power, and 5 mTorr pressure (process #11 in Table S1; see Figure S11 for nanohole arrays with a diameter-to-depth ratio of up to 1.7 fabricated on SiO_2_ using the same plasma conditions). The nanopatterns in PPA are accurately and proportionally transferred into the dielectric layers with a significant increase in amplitude and without adding extra roughness during dry etching, keeping the pattern depth-to-roughness ratio almost constant. This is achieved due to the high vertical etch rates with respect to lateral etch rates. In Fig. 3, we also show Fourier transforms of the measured topographies on PPA and SiO_2_ to evaluate the quality of the pattern transfer. Fourier transforms are performed on the measured topographies along 140 lines (2.8 x 2.8 *μ*m^2^ area with 20 x 20 nm^2^ pixels) and then averaged. The second harmonic has an amplitude of 3.1% of the main Fourier component at the spatial frequency of 2.5 *μ*m^−1^ after t-SPL patterning on PPA, whereas the one measured after pattern transfer and amplification in SiO_2_ has an amplitude of 3.4% of its main Fourier component for sinusoidal patterns amplified from 20 ± 1 nm to 102 ± 2 nm peak-to-peak depths (Fig. 3e, f). This indicates that a 5-fold aspect ratio amplification is achieved without any significant distortion of the original sinusoidal shapes. At higher depth amplifications, distortion of the sinusoidal shapes becomes significant. For instance, for 12 W RF bias power, a 10 times depth amplification of the sinusoidal nanopatterns is obtained. However, the sinusoidal shapes are deformed because of increased lateral etching, with a second harmonic component as large as 37% of the main component (see Figures S12–S14 for AFM images and Fourier transform).

### Application of grayscale stamps to nanoimprint lithography

**Fig. 4:**
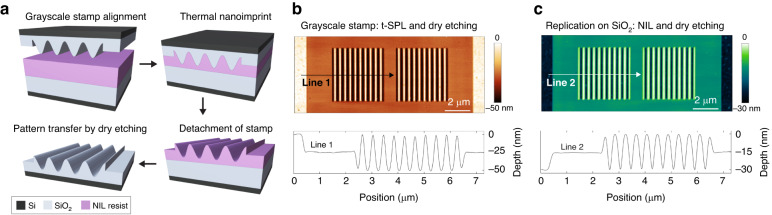
NIL and grayscale pattern transfer. **a** Thermal NIL of grayscale nanostructures on thermoplastic resist and pattern transfer into SiO_2_ substrates. **b** AFM image of grayscale stamp fabricated on thin film SiO_2_ by combining t-SPL and dry etching. **c** AFM image of nanopatterns replicated on thermoplastic resist and transferred into a SiO_2_ dielectric layer with CHF_3_/SF_6_ plasma (see Figure S16 for AFM image of replicated nanostructures on NIL resist)

In the following, we discuss the combination of t-SPL with dry etching for grayscale NIL stamp fabrication as a possible strategy to overcome the limited throughput of t-SPL^41^. NIL is a cost-efficient technique to replicate high-resolution grayscale nanostructures on large surfaces by step-and-repeat process^42,43^. We use a structured SiO_2_ (500 nm thick)/Si substrate fabricated by t-SPL and dry etching processes shown above as grayscale stamp to replicate the nanopatterns on a thermoplastic NIL resist, mr-I 8010R, and then transfer them into thin dielectric films on wafers. The fabricated 50 nm in depth sinusoidal nanostructures on SiO_2_ with surface roughness of 0.5 nm_rms_ are replicated on a 100 nm thick thermoplastic resist layer with surface roughness of 0.5 nm_rms_. Then, the nanopatterns are transferred from the NIL resist to a SiO_2_ thin film by dry etching (Fig. 4) using CHF_3_/SF_6_ (50/15 sccm) plasma (see Methods). These transferred sinusoidal nanopatterns on 100 mm wafer have 30 nm depths with surface roughness of 0.3 nm_rms_, proportional to the depth reduction (NIL resist to SiO_2_ etch selectivity is 1:0.6). The integration of t-SPL, NIL, and dry etching techniques demonstrated in this work is suitable for scalable and reproducible fabrication of high-resolution grayscale nanopatterns.

### Application of grayscale nanostructures to strain 2DMs

**Fig. 5:**
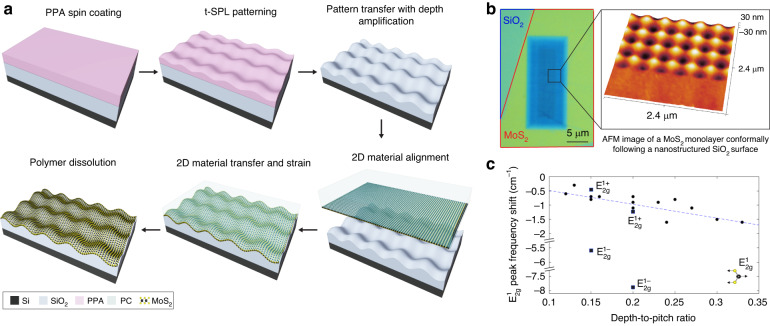
Local strain nanoengineering of 2DMs. **a** Fabrication process flow of grayscale dielectric nanostructures for 2DMs strain. **b** Optical microscope (left) and AFM images of a MoS_2_ monolayer that conformally covers the SiO_2_ layer nanostructured with sinusoidal waves modulated in two dimensions (400 nm pitch, 60 nm peak-to-peak depth). **c** Raman micro-spectroscopy to visualize strain modulation of MoS_2_ monolayer on grayscale SiO_2_ nanostructures having different depth-to-pitch ratios (pitches from 300 to 500 nm, and depths from 60 to 120 nm). The plot shows the shift of ${\,{{\mbox{E}}}\,}_{2g}^{1}$ Raman peaks, ${\,{{\mbox{E}}}\,}_{2g}^{1-}$ and ${\,{{\mbox{E}}}\,}_{2g}^{1+}$ peaks in case of peak splitting, as a function of depth-to-pitch ratio. Raman peaks of unstrained MoS_2_ are obtained from the part of the flake placed on flat SiO_2_ surfaces

Similar to the approach used in Si-based devices^44^, the strain has been used for 2D materials (2DMs) to increase the charge carrier mobility in field-effect transistors^45^. Recently, we used t-SPL to obtain strain-induced bandgap modulation of 2DMs placed on top of a PPA layer with thermomechanical indentations^46^. Here, we fabricate grayscale dielectric nanostructures with higher aspect ratios, switching from PPA to high-quality SiO_2_ as the underlying dielectric. This provides a robust platform for straining 2DMs. The increase in aspect ratio is translated into a higher strain, and the absence of the underlying PPA polymer reduces the risk of a possible degradation of the nano-corrugations over time. In this way, we induce deterministic local strain to 2DMs through the precise nanopatterning of the substrate in contrast to other cases where only random surface roughness^45^ or global strain^47^ is used. We fabricated a SiO_2_ layer with sinusoidal waves modulated in two dimensions (*f*(*x*, *y*) = *A* [$\cos (gx)$ + $\cos (gy)$]). MoS_2_ monolayer grown by metal-organic chemical vapor deposition (MOCVD)^48^ was then transferred by pressing against the grayscale nanostructures using a polymer support (Fig. 5a; see Methods). This induces a bi-axial tensile strain to the 2DMs deterministically placed on structured SiO_2_ layers.

Compared to other studies with nanopillar arrays fabricated by EBL^49^, our sinusoidal pattern avoids sharp edges that pose a high risk of breaking the 2DMs and allows for conformal attachment of the 2DM to the nanopatterned dielectric layer through van der Waals forces, mitigating wrinkling and suspension of the 2DMs (Fig. 5b). The use of t-SPL is consequently a promising option to produce mechanically stable semiconductor-dielectric interfaces for strained 2DMs-based devices (Figure S17). In addition to high-resolution AFM imaging, the continuity of 2DM flakes and their conformal adherence to the structured substrate are further studied by TEM and energy-dispersive X-ray (EDX) elemental mapping (Figure S18). Based on the analysis, we conclude that the flakes are intact on sinusoidal nanopatterns. When MOCVD-grown MoS_2_ monolayers are strained on grayscale nanostructures, both the ${\,{{\mbox{E}}}\,}_{2g}^{1}$ and A_1*g*_ modes exhibit redshifts. These shifts correspond to the optically averaged strain induced over about 1 *μ*m^2^ area of the atomically thin material^50^. Different strain values in the same 2DMs are obtained by changing the depth-to-pitch ratios of the nanostructures, as shown in Fig. 5c. The linear fit of the Raman measurements shows about -5 cm^−1^ shift per unit dept-to-pitch ratio for ${\,{{\mbox{E}}}\,}_{2g}^{1}$ modes. In some cases, up to 7.9 cm^−1^ redshifts, corresponding to a 1.8% strain according to previously reported experiments^51^, are observed. The smaller redshifts are most probably caused by the formation of the cracks during the two-step manual handling process, which causes strain relaxation (see Methods; Table S2; Figures S19 and S20). Although with large deviations, this data indicates, as intuitively expected, that higher depths in sinusoidal nanopatterns result in higher tensile strain in 2DMs.

## Conclusion

In this work, we present an innovative fabrication process for grayscale nanopatterning, specifically with depths greater than 100 nm and up to 400 nm, based on the combination of t-SPL and dry etching. Thanks to the high etch selectivity of SiO_2_ and Si_3_N_4_ compared to PPA in optimized CHF_3_/SF_6_ RIE conditions with controlled substrate temperature, this process enables the fabrication of complex high aspect ratio nanostructures. Depth amplifications up to 5 times into SiO_2_ of the shallow polymer patterns written by t-SPL were achieved without introducing shape distortion and additional surface roughness due to plasma process. A 10-fold amplification is also achieved, although with significant distortion from the sinusoidal shape. We introduce a cycled process alternating between plasma etching and cooling steps to prevent substrate overheating, influencing, in turn, the aspect ratio amplification.

To exemplify the possible applications of the proposed process, we show its applications to NIL and to strain engineering of 2DMs. The grayscale nanostructured substrates are used as a stamp for thermal NIL to address the scalability limitation of scanning probe-based fabrication. Sinusoidal nanosurfaces are faithfully replicated in dielectric films by combining NIL and dry etching. The developed etch recipes were utilized for pattern transfer from PPA to SiO_2_ and Si_3_N_4_, as well as from thermoplastic NIL resist to SiO_2_, demonstrating consistent quality, particularly in terms of roughness. We also present the use of sinusoidal dielectric layers as a tool for 2DM strain engineering, where the amplitude modulation of the sinusoidal waves is tuned to control the local strain rates. Bi-axial tensile strains are achieved in the same flake of MoS_2_ monolayer on structured areas. Varying depth-to-pitch ratios of the nanostructures are used to control locally the induced strain in atomically thin material. This opens the way to use the high aspect ratio and smooth dielectric grayscale nanopatterns presented in this study for the development of advanced nanoelectronics, photonics, and optoelectronics devices, with potential applications in sensors and processors.

## Materials and Methods

### Sample preparation and grayscale nanopatterning

For rectangular and sinusoidal ($f(x,y)=A\cos (gx)$) nanopattern fabrication, a 5 wt% solution of polyphthalaldehyde (PPA, Allresist) in anisole (Sigma-Aldrich Chemie GmbH) was spin-coated on SiO_2_ (500 nm thick wet oxide)/Si (500 *μ*m thick, 10 x 10 mm^2^ size) substrate or Si_3_N_4_ (low-pressure chemical vapor deposited (LPCVD) 500 nm thick)/Si (500 *μ*m thick, 10 x 10 mm^2^ size) substrate at 5000 rpm and soft baked at 110 °C for 2 min. For sine waves (*f*(*x*, *y*) = *A*[$\cos (gx)$ + $\cos (gy)$]) fabrication, a 5 wt% PPA solution in anisole was spin-coated on SiO_2_ (200 nm thick dry oxide)/Si (500 *μ*m thick, 10 x 10 mm^2^ size) substrate at 5000 rpm and soft baked at 110 °C for 2 min.

A commercial t-SPL system (Nanofrazor Explore) and thermal cantilevers of type NanoFrazor Monopede (Heidelberg Instruments Nano AG) were used to pattern grayscale nanostructures on PPA. For grayscale design, the analytical design of a sinusoidal surface was converted into a grayscale bitmap consisting of a 20x20 nm^2^ pixel grid. The normalized depth was set to 256 levels. Then, the grayscale bitmap image was imported into the t-SPL software, and the depth for each pixel was assigned. In case of combined binary and sinusoidal design (Figure S1), the minimum depth (white pixel) and maximum depth (black pixel) were set to 10 nm and 40 nm, respectively. For t-SPL, the writing heater temperature was set to 950 °C, and the step size to 20 nm, the scan speed to 25 *μ*s per pixel, and the force pulse to 5 *μ*s. The patterned depths were simultaneously corrected by integrated in-situ AFM metrology, and NanoFrazor’s Kalman feedback system adjusted the actuation forces for high-resolution depth control.

### Reactive ion etching

The nanopatterns were transferred from PPA to thin film SiO_2_ by a commercial ICP-based RIE system (SPTS Advanced Plasma System). In the dry etching processes, high density CHF_3_/SF_6_ and CHF_3_/SF_6_/O_2_ plasma were used at different flow rates, plasma times, process pressures and RF bias powers applied to the bottom electrode for wafer voltage biasing (independent from the RF ICP source), but at fixed RF ICP power of 950 W (13.56 MHz RF field). 10 x 10 mm^2^ size substrates were glued onto 100 mm silicon wafers by mounting wax (PELCO® Quickstick 135). The wafer was placed on the bottom electrode and was gripped by electrostatic clamping. A backside cooling flow of He was used to cool down the substrate temperature to 10 °C. For the processes lasting longer than 100 s, the plasma was turned off to lower the substrate temperature. Meanwhile, Ar was inserted into the plasma chamber to accelerate substrate cooling. At the end of the dry etching processes, substrates were removed from the wafer by melting the mounting wax on a hot plate at 135 °C, and cleaned with 5 min acetone, 5 min IPA and 10 min O_2_ plasma. For cleaning of the substrates used for 2DM strain, a piranha solution (3:1 mixture of H_2_SO_4_(96%):H_2_O_2_(30%)) was used for 10 min followed by 5 min acetone, 5 min IPA and 10 min O_2_ plasma.

### Nanoimprint lithography and pattern transfer

For thermal NIL replication on thermoplastic resist, mr-I 8010R resist (micro resist technology GmbH) was spin-coated on SiO_2_ (500 nm thick wet oxide)/Si (500 *μ*m thick) 100 mm wafer at 3000 rpm and soft baked at 90 °C for 1 min. Grayscale stamps fabricated by combining t-SPL and dry etching were placed on mr-I 8010R resist-coated wafer. NanoImprint EHN-3250 thermal nanoimprinter was used for the replication of grayscale nanostructures. 0.2 MPa pressure was applied at 160 °C for 5 min and cooled down to 90 °C in 1 min. Demolding of the grayscale stamp was performed at 90 °C. Grayscale nanostructures on mr-I 8010R resist were then transferred to SiO_2_ thin films on Si wafers using the same ICP-based RIE system (SPTS Advanced Plasma System) with CHF_3_/SF_6_ plasma with a flow rate of 50/15 sccm at 950 W RF ICP power, 80 W RF bias power, 5 mTorr pressure, and 10 °C substrate temperature.

### 2D material growth and transfer

The MoS_2_ flakes are grown on a c-plane sapphire chip by the MOCVD, as presented by Cun et al.^48^. The sapphire chip was annealed in air for 6 hours to achieve a smooth atomic surface and then spin-coated with 0.026 mol/L NaCl solution in deionized water to suppress nuclear density and accelerate the growth rate. Then, the chip was loaded into a tube furnace with controlled temperature and gas flow rate. During the growth process, molybdenum hexacarbonyl (Mo(CO)_6_) and hydrogen sulfide (H_2_S) were introduced into the quartz tube as precursors using Ar as the carrier gas. The flow rates of Mo(CO)_6_ and H_2_S were set at 6 sccm and 3 sccm, respectively. The Mo(CO)_6_ precursor was stored in a bubbler immersed in a water bath maintained at a temperature of 15 °C to achieve a constant vapor rate. Small amounts of H_2_ and O_2_ were separately introduced into the growth chamber to balance the growth rate and achieve MoS_2_ monolayers. At the end of the growth, the precursor supply was abruptly stopped, and the furnace was allowed to cool naturally to room temperature with a flow of 200 sccm of Ar to remove gaseous residues.

The sapphire chip with MoS_2_ monolayers was then spin-coated with 50 nm thick PMMA, and 1 mm thick PDMS film was placed on top. The PDMS/PMMA/2D flakes stack was detached from the sapphire chip after 20 min immersion in DI water and was transferred to SiO_2_ (270 nm thick)/Si (500 *μ*m thick) chips. The polymer layers were then removed in acetone. The SiO_2_/Si chip was heated to 95 °C, and the MoS_2_ monolayer flakes were picked up with a polycarbonate (PC) film, which was mounted on a curved PDMS layer attached to a glass microscope slide. Later, these flakes were aligned under a microscope on grayscale nanostructures fabricated by t-SPL and dry etch-based transfer and pressed against the grayscale nanostructures. Flakes were transferred with the PC film layer after heating the substrate to 180 °C. The PC film was later dissolved in chloroform for 1 hour.

### AFM, SEM and Raman spectroscopy

In addition to in-situ metrology of written patterns during t-SPL, AFM topography characterization was performed with Bruker FastScan AFM (ScanAsyst mode). ScanAyst auto control was used as a feedback system, and the step size in topography imaging was set to 20 nm. To compare the average peak-to-peak depth amplifications, AFM topography characterization on patterned SiO_2_ or Si_3_N_4_ was performed with Bruker FastScan AFM (ScanAsyst mode, ScanAyst auto control feedback, and step size of 20 nm) in the same way as for PPA. For data visualization and surface profile characterization, the scanning-probe analysis software Gwyddion (version 2.59) was used. Data plotting and Fourier transforms of patterns were performed in MATLAB (version R2020b). RMS surface roughnesses on flat areas are quantified according to the mean value of the region of interest. RMS surface roughnesses on sinusoidal areas are calculated by substracting the mean sinusoidal profile of the measured topographies over 140 lines in 2.8x2.8 *μ*m^2^ area with 20 x 20 nm^2^ pixels.

AFM images on 2DMs were taken in PeakForce QNM® mode using the Multimode (Bruker) Scanhead and Nanoscope V controller (Bruker). ScanAsyst-Air cantilevers with a spring constant of 0.4 N/m were used, and peak forces were set to 350 pN. Quantitative mechanical characterizations were performed with 10 nN peak force setpoints.

The cross-sections of the final structures were examined with a dual-beam Focused Ion Beam/Scanning Electron Microscope (FIB/SEM) instrument (Zeiss CrossBeam 540). SEM images were obtained by InLens secondary-electron (SE) detector at 2 kV electron high tension (EHT), 5 mm working distance, and 300 pA probe current. Images were taken at 54° stage tilt and tilt correction was activated during imaging.

For the Raman spectra collection, a confocal Raman microscope system (inVia Qontor, Renishaw) coupled with an Olympus inverted optical microscope was utilized. The Raman spectra was collected by averaging 2 accumulations of 10 s laser exposure with an excitation wavelength of 532 nm. A grating of 3000 gr/mm was used for Raman characterization. To avoid damaging of the studied samples, the laser power was kept lower than 100 *μ*W. The peak positions are extracted by fitting the curves with Lorentzian functions.

### Supplementary information

Supplementary Information
