# Supplementary material for: Combining thermal scanning probe lithography and dry etching for grayscale nanopattern amplification
Source: Microsyst Nanoeng. 2024 Feb 23;10:28. doi: 10.1038/s41378-024-00655-y (PMC10891065; doi:10.1038/s41378-024-00655-y)
Supplement: Supplementary file 1 — Supplementary Information [file 41378_2024_655_MOESM1_ESM.pdf]

SUPPLEMENTARY INFORMATION

Combining thermal scanning probe lithography and dry etching for grayscale nanopattern amplification

Berke Erbas, Ana Conde-Rubio, Xia Liu, Joffrey Pernollet, Zhenyu Wang, Arnaud Bertsch, Marcos Penedo, Georg Fantner, Mitali Banerjee, Andras Kis, Giovanni Boero, Juergen Brugger

Section 1: Combination of t-SPL and dry etching

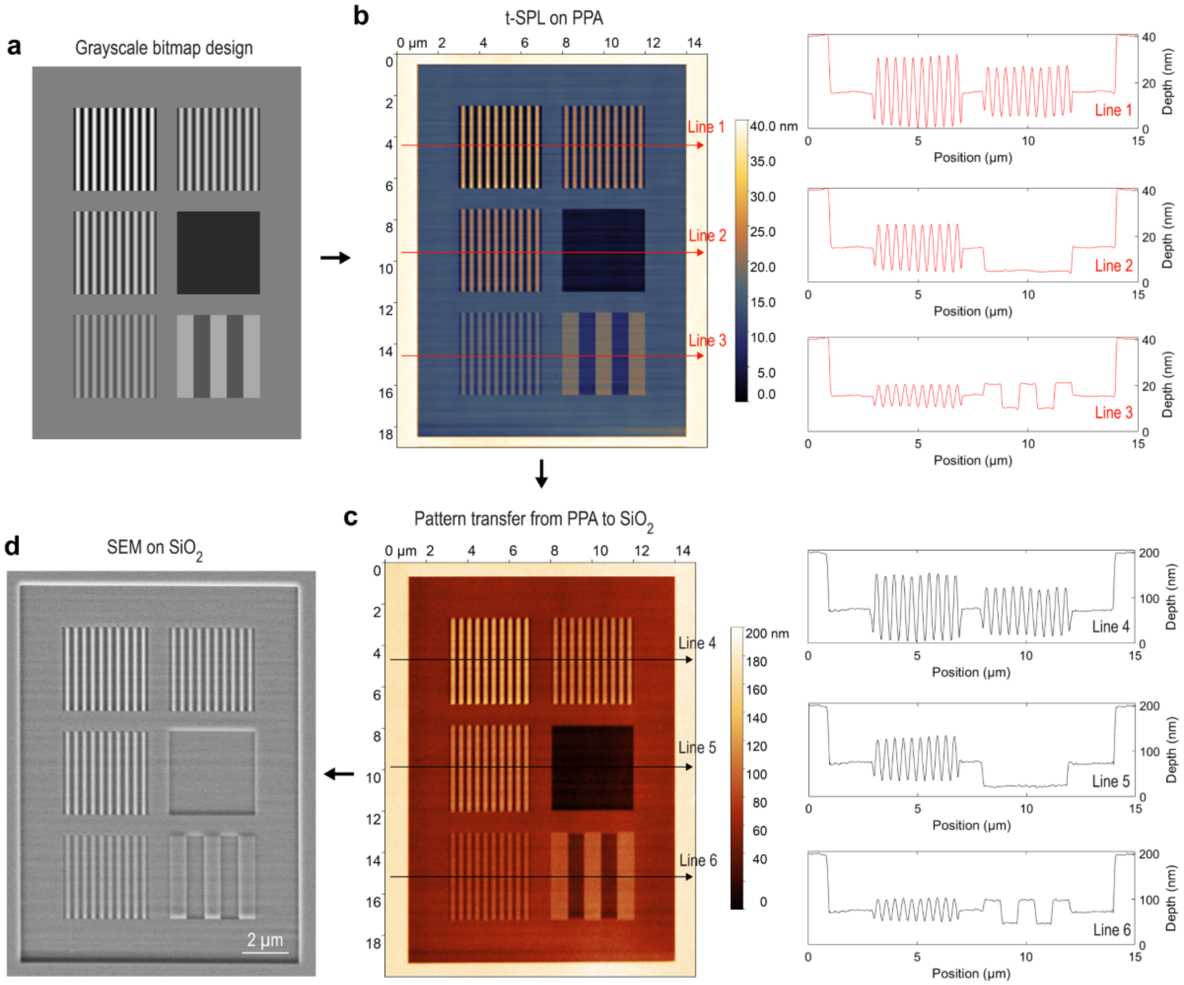

Figure S1: Grayscale nanopatterning of dielectrics. (a) Grayscale bitmap having a 20x20 nm^2 pixel grid with 256 levels of normalized depth. (b) AFM image of rectangular and sinusoidal nanopatterns having different peak-to-peak depths fabricated on PPA by t-SPL, with depths defined for each pixel on the grayscale bitmap in (a). (c) AFM image of grayscale nanostructures anisotropically transferred from PPA to SiO_2 with peak-to-peak depth amplification. (d) 30° tilted SEM image of patterns transferred into SiO_2 after dry etch transfer.

Section 2: Patterning with thermal scanning probe

The tip geometry strongly affects the spatial resolution during patterning. The conical shape of the tip defines the written pattern size. Deeper indentations cause broader patterns. In addition, during lithography, the scanning probe cantilever has an inclination angle of 5±1° , causing non-symmetrical and wider conical openings on PPA for deeper indentations (Figs. S3 and S4). As the wider part of the hot tip plays a role in heat transfer, the spatial resolution decreases, which can be detrimental when fabricating closely spaced patterns. Consequently, the inherent properties of scanning probe-based fabrication limit the aspect ratios of the fabricated nanostructures.

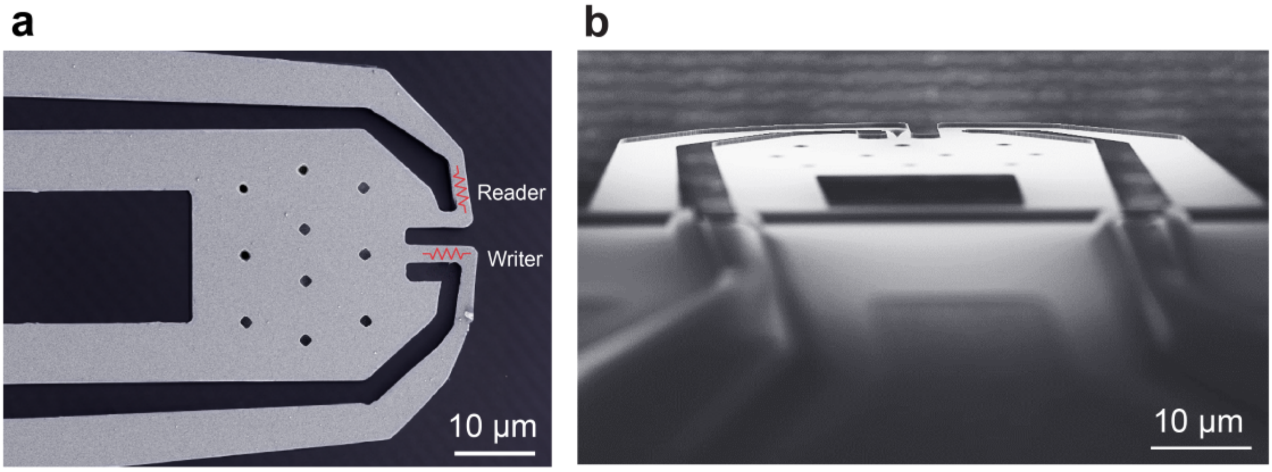

Figure S2: SEM images of the thermal scanning probe. (a) The top view and (b) tilted view images taken with a SEM of a commercial silicon thermal cantilever (NanoFrazor Monopede, Heidelberg Instruments Nano AG).

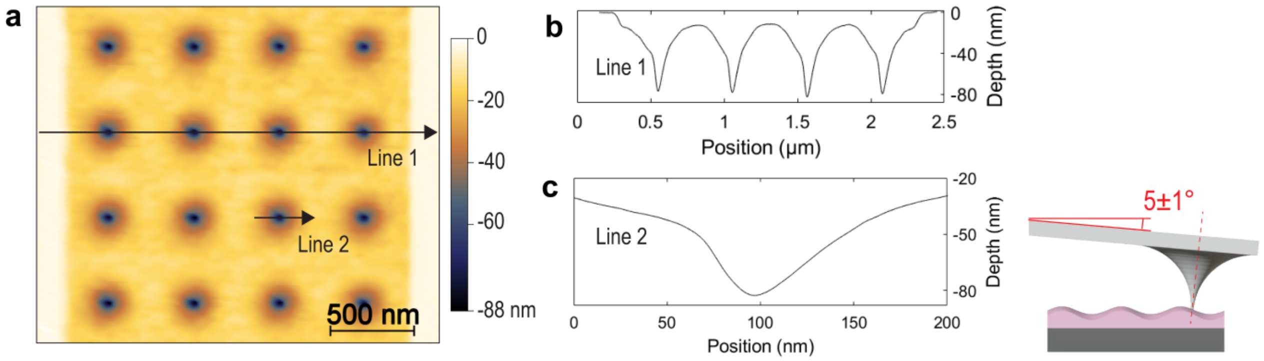

Figure S3: Experiment for t-SPL patterning depth and lateral resolution determination. (a) *In-situ* AFM image of t-SPL patterns having deep indentations. (b) Cross-section profile obtained along Line 1. The depth of patterning is limited due to tip cooling for deep indentation. (c) Cross-section profile taken along Line 2. As the angle of scanning probe during writing is 5±1°, the patterning is non-symmetric. The shape of the written patterns on PPA are defined by the conical shape of the scanning probe tip, and the minimum feature size patterned is comparable to the tip diameter.

While patterning sinusoidal shapes with depths up to 50 nm, t-SPL results in high-resolution patterning. However, for writing depths in the order of 100 nm and above, tip patterning deviates from the targeted design due to increased tip cooling and decreased heating efficiency. These effects result in non-uniform depths even after depth feedback correction on the Nanofrazor tool, which adjusts the actuation forces for depth control. Patterning for a depth exceeding 100 nm leads to rough and deformed surfaces, making it impractical for our purpose (Figs. S4, S5, and S6).

Larger tip deflection, higher electrostatic tip actuation, increased tip temperature, and longer contact time between the tip and polymer are required to achieve higher indentation depths in the polymer. Nevertheless, the tip geometry limits the patterning of sinusoidal nanostructures with pitches below 1 µm. Additionally, the maximum achievable patterning depth is constrained by cantilever deflection, typically in the range of a few hundred nanometers. However, the quality of written polymer structures is influenced by tip shape and heat transfer efficiency.

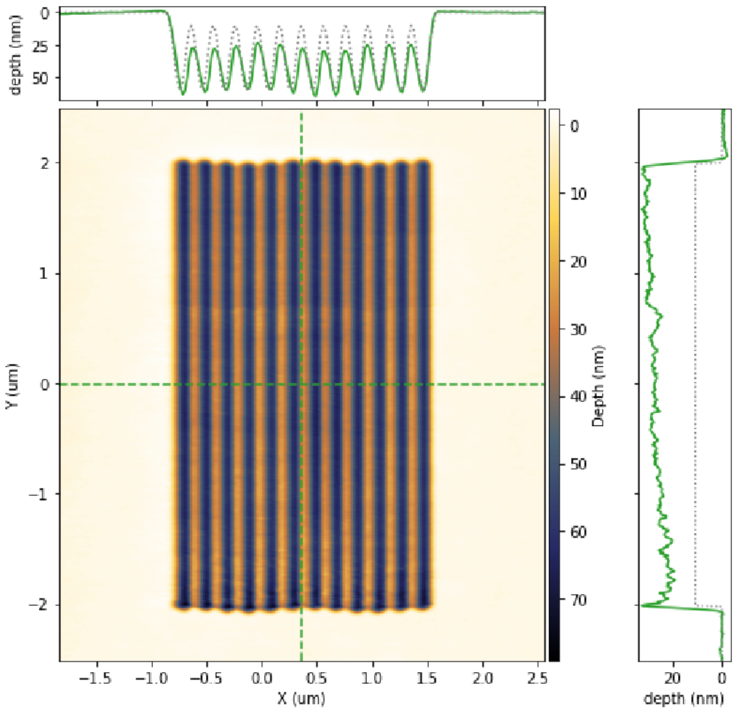

Figure S4: The aspect ratio limitation of grayscale nanopatterns written by t-SPL. For patterning depths exceeding 50 nm, the conical geometry of the tip hinders the fabrication of closely spaced patterns and imposes limitations on the aspect ratios of the engineered nanostructures, such as sinusoidal nanopatterns with a pitch of 200 nm. The green lines depict the written surface profiles, and the dashed lines represent the targeted depths.

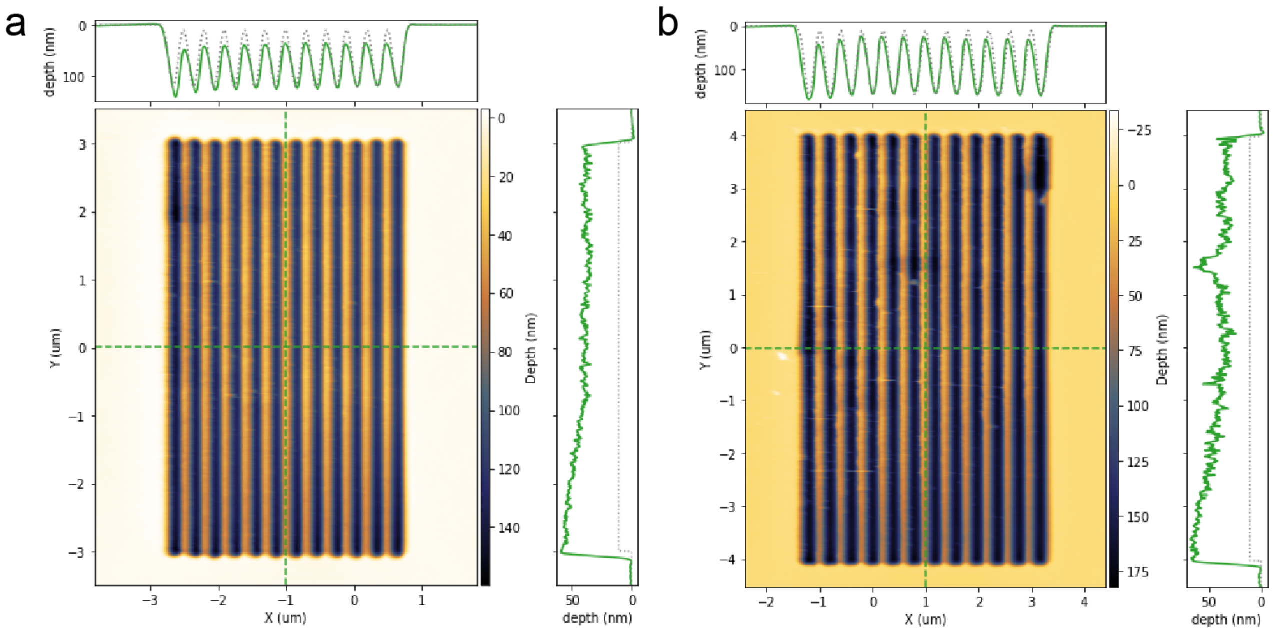

Figure S5: The depth and aspect ratio limitation of grayscale nanopatterns written by t-SPL. *In-situ* AFM images of (a) sinusoidal nanopatterns with a pitch of 400 nm and a maximum depth of 110 nm and (b) sinusoidal nanopatterns with a pitch of 400 nm and a maximum depth of 140 nm show that patterning depths above 100 nm are not practical and cause rough and deformed surfaces. Increased penetration into the resist results in a decline in heating efficiency due to tip cooling. The green lines depict the written surface profiles, and the dashed lines represent the targeted depths.

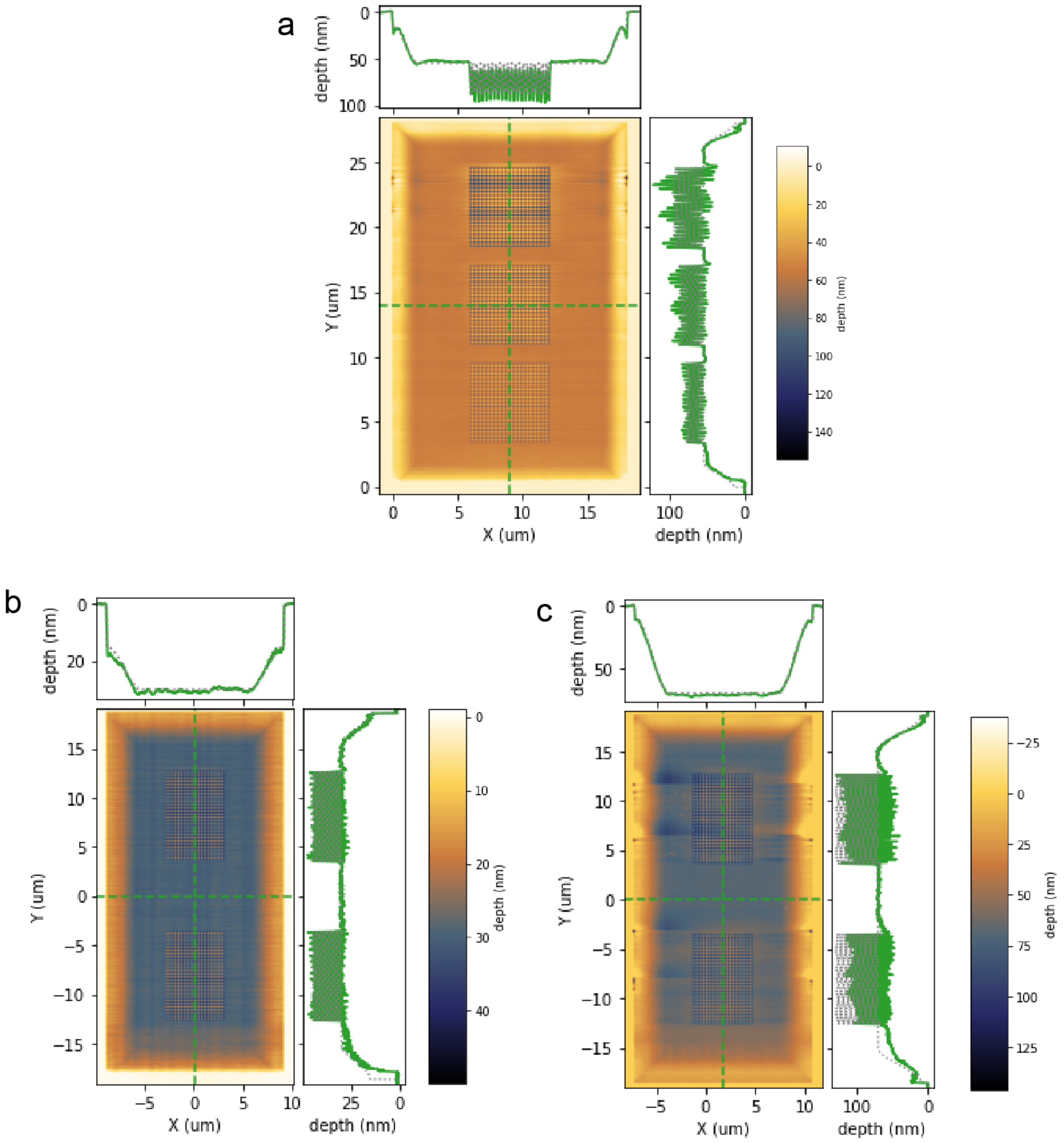

Figure S6: The depth limitation of t-SPL patterning. (a) Sinusoidal waves modulated in two dimensions with a pitch of 300 nm and varying amplitudes. For depths of 100 nm and above, tip patterning deviates from the targeted design, resulting in non-uniform depths even after depth feedback correction, which adjusts the actuation forces for high-resolution depth control. Additionally, sinusoidal waves modulated in two dimensions with a pitch of 300 nm exhibit (b) a maximum depth of up to 50 nm and (c) up to 120 nm. It is evident that patterning for depths exceeding 100 nm is impractical.

Section 3: Effect of pressure on depth amplification

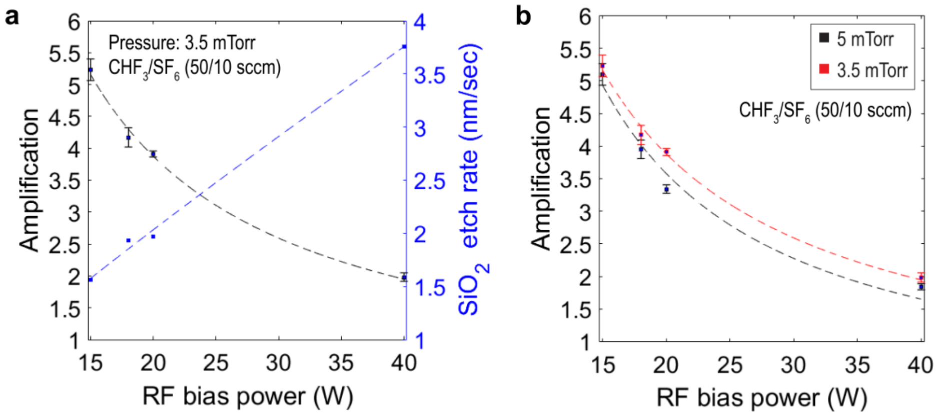

Figure S7: Pattern transfer from PPA to SiO_2. (a) The effect of RF bias power on depth amplification and SiO_2 etch rate for the recipe containing CHF_3/SF_6 (50/10 sccm) gases at 3.5 mTorr pressure. (b) Comparison of the effect of different plasma pressure (5 and 3.5 mTorr) on depth amplification. The error bars indicate one standard deviation ($\pm\sigma$) and the dashed lines are exponential fits of the experimental data.

Section 4: Pattern transfer to Si_3N_4

CHF_3/SF_6 plasma is also used to provide a depth amplification of up to 2.1 ± 0.1 while transferring the patterns from PPA to Si_3N_4 thin films, but with reduced selectivity compared to SiO_2. For RF bias power lower than 18 W, the impact of physical etching that contributes to depth amplification is mitigated, and the vertical depth amplification starts to decline in Si_3N_4 thin films. See Figure S8 for Si_3N_4 etch rates at different RF bias powers of 14, 15, 18, 20, and 40 W.

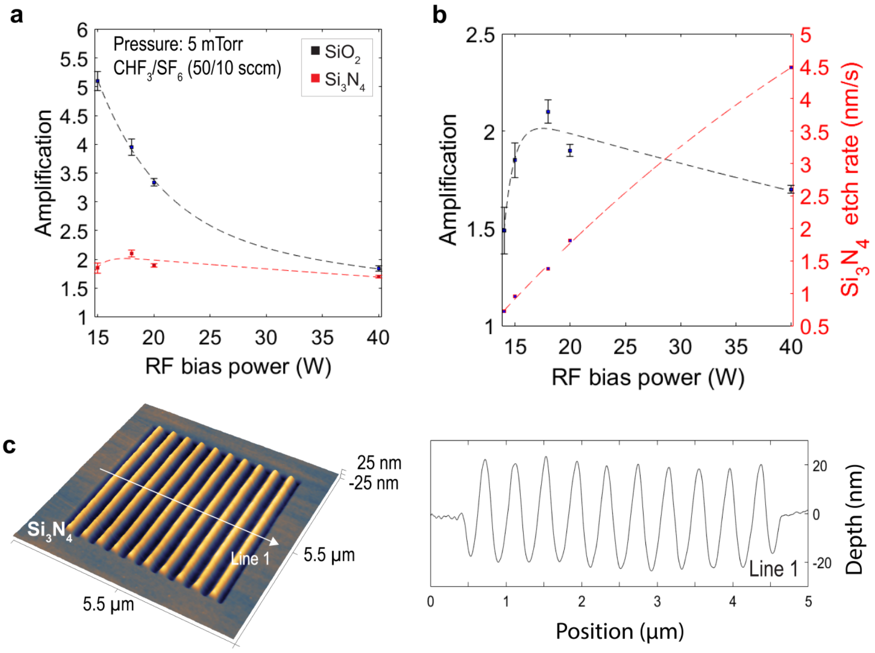

Figure S8: Pattern transfer from PPA to Si_3N_4. (a) Comparison of SiO_2:PPA and Si_3N_4:PPA depth amplifications at different RF bias powers for the same plasma conditions. (b) Effect of RF bias power on depth amplification and Si_3N_4 etch rate for CHF_3/SF_6 plasma with flow rate of 50/10 sccm at 5 mTorr fixed pressure. The error bars correspond to one standard deviation ($\pm\sigma$) for 16 surface profile comparisons, and the dashed lines are polynomial fits. (c) AFM image of transferred sinusoidal nanopattern on Si_3N_4 after 2.1 times grayscale depth amplification and cross-section surface profile (Line 1).

Section 5: Substrate cooling during plasma etching

To prevent substrate overheating during dry etching, we use a cycled RIE process that alternates between plasma etching and cooling phases. With continuous plasma etching, sinusoidal nanopatterns having 20 nm peak-to-peak depths are transferred from PPA to SiO_2 in 300 s with a depth amplification of 4 times using CHF_3/SF_6 plasma with flow rate of 50/10 sccm at 950 W RF ICP power, 15 W RF bias power, and 5 mTorr pressure (process #1 in Table S1). When the dry etching process is paused every 100 s of plasma etching to let the substrate cool, the average grayscale depth amplification is increased by up to 33% compared to continuous plasma etching. Experimental results show that under identical plasma parameters, varying cooling durations of 2, 5, 10, and 15 min following 100 s of plasma etching result in average depth amplifications of 4.5, 5.1, 5.1, and 5.2, respectively. This indicates that the substrate cools down to a temperature close to the initial substrate temperature in about 5 min when the plasma is off (see Fig. S9b,c). Higher temperatures affect the sidewall protection, causing the etching of the top parts of sinusoidal patterns and, in turn, reducing the depth amplification. Thus, shorter plasma etching times provide higher amplifications. The plasma time is fixed at 100 s, as no significant increase in depth amplification is observed below 120 s, as shown in Fig S9a (an increase of 24% is observed between 300 and 120 s of plasma etching, compared to only 4% between 120 and 100 s).

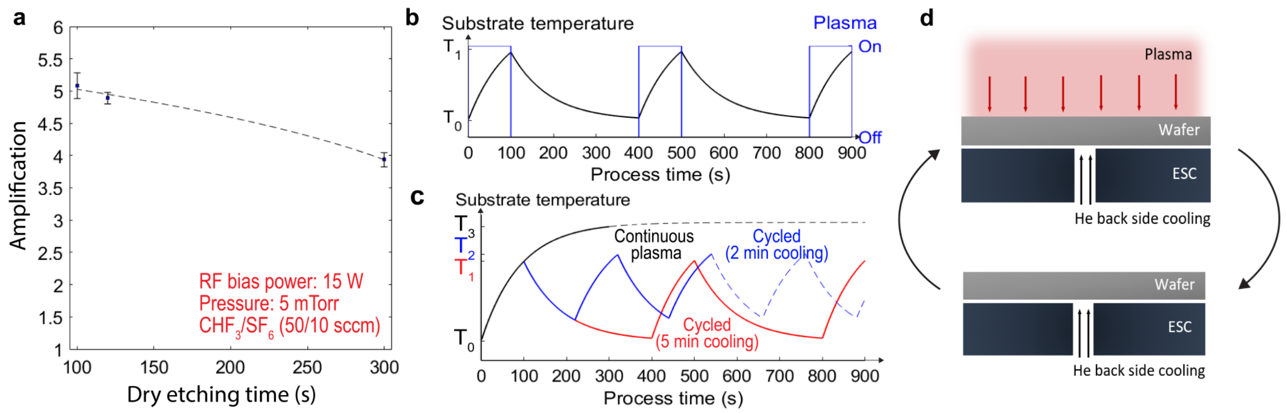

Figure S9: Effect of substrate temperature. (a) Comparison of continuous plasma etching with cycled 120 s and 100 s of plasma etching followed by 5 min cooling in between plasma processes. (b) Effect of cycled plasma etching and cooling process on wafer surface temperature. (c) Graphical demonstration of the wafer surface temperature hypothesis. While T_0 represents the initial substrate temperature, T_3 represents the wafer surface temperature after 300 s of continuous plasma etching. T_1 and T_2 represent the highest surface temperatures achieved during the cycled etching processes with a 5 min and 2 min cooling period between 100 s of plasma etching processes, respectively. The maximum substrate surface temperatures achieved after 300 s of continuous plasma (T_3). When the same thermal time constants are considered, continuous dry etching causes the highest substrate temperature, and the dry etching process with 2 min cooling has a higher temperature than 5 min cooling ($T_2 > T_1$). The dry etch recipes and the results of dry etch transfer are listed in Table S1. (d) Cycled plasma etch and cooling process illustration. Cooling is provided by the wafer backside cooling system of the ICP-RIE machine and allows lowering the wafer surface temperature when the plasma is off.

Section 6: Surface roughness of grayscale nanostructures

Cheong et al. [1] achieved pattern transfer from PPA to SiO_2 with selectivity 1:2 by using CHF_3 plasma. Rawlings et al. [2] successfully transferred sinusoidal structures patterned on PPA by t-SPL into SiO_2 with a selectivity of 1:0.96 by using C_4F_8/SF_6 plasma. Following this grayscale pattern transfer demonstration, Lisunova et al. [3] studied different plasma etching processes to amplify the shallow t-SPL patterns on PPA, achieving up to 3-fold depth amplification while transferring patterns from PPA to SiO_2 thin films by C_4F_8/ H_2/He plasma. However, in this plasma etching process, the surface roughness increased up to 8 times (from 0.3 nm_{rms} on PPA to 2.3 nm_{rms} on SiO_2).

We experimentally demonstrate that the CHF_3/SF_6 plasma recipe with a flow rate of 50/10 sccm at 950 W RF ICP power and 5 mTorr pressure does not show any significant increase in roughness after pattern transfer from PPA to SiO_2 (Fig. S10). The surface roughness is quantitatively characterized by measuring the RMS roughness of both sinusoidal surfaces and flat parts, yielding similar values. The amplification in surface roughness on the SiO_2 surface is similar to the depth amplification of the sinusoidal nanopatterns. In the ideal case of no additional roughness caused by plasma etching, the ratio of depth amplification to roughness increase would be equal to 1. The methodology presented in this work results in a value larger than 0.92 for amplifications larger than 2 times. The remarkable lateral etching of nanopatterns smoothes sharp nanopeaks, resulting in a lower surface roughness increase than the depth amplification (ratio of 1.08, Fig. S10c). While depth amplification comes from the higher etch rate in the vertical direction compared to the lateral one, it is important to note that lateral etching cannot be entirely eliminated. Longer total etching time leads to increased lateral etching, resulting in the smoothing of these peaks.

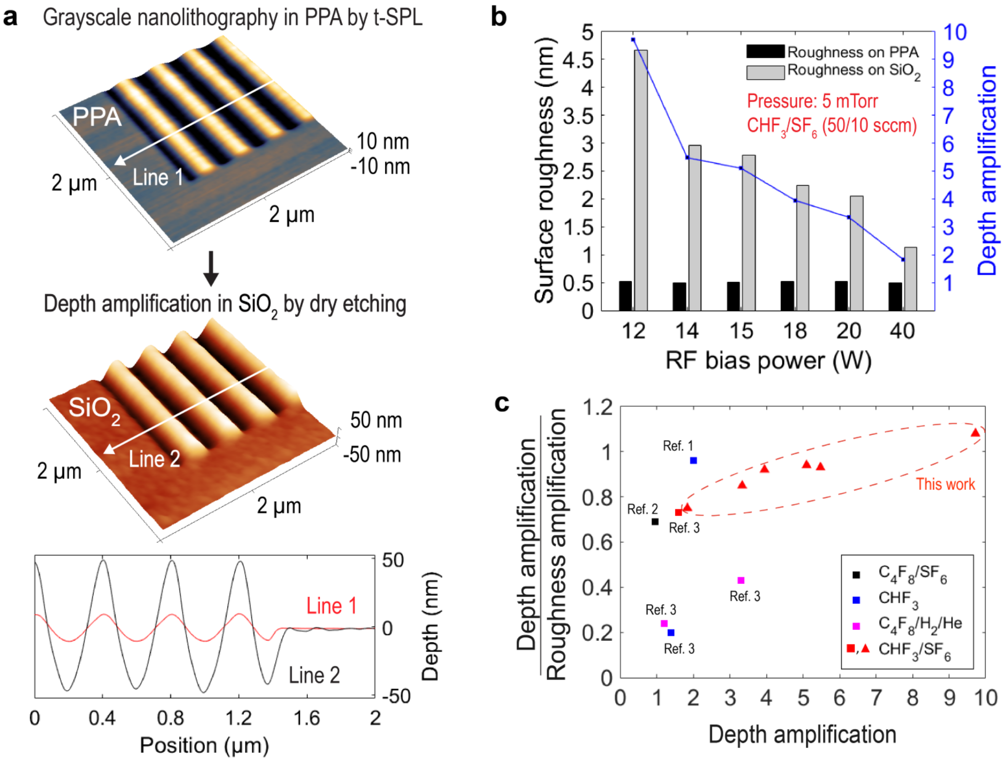

Figure S10: Effect of depth amplification on surface roughness. (a) AFM images and amplitude comparison of a sinusoidal pattern before (Line 1) and after (Line 2) dry etch transfer that shows no major additional roughness after dry etching (CHF_3/SF_6 plasma with flow rate of 50/10 sccm at 950 W RF ICP power, 15 W RF bias power, and 5 mTorr pressure). (b) Comparison of surface roughness on written patterns on PPA (black) with t-SPL and transferred (gray) patterns, and the depth amplification of nanopatterns after dry etching obtained for varying values of RF bias power. During pattern transfer, the roughness of the structures undergoes the same amplification as the structures themselves. (c) Comparison of several dry etching recipes for pattern transfer from PPA to SiO_2 based on their depth amplification over roughness amplification ratio.

Section 7: Nanohole array fabrication by t-SPL and dry etching

We fabricate nanohole arrays with a diameter-to-depth ratio of up to 1.7 (Fig. S11). Shallow depth patterns on PPA are amplified in SiO_2 due to the high etch selectivity of SiO_2 compared to PPA. These grayscale and binary structures in SiO_2 can be used as etch-resistant hard masks, and pattern depths can be further increased in Si. Therefore, the combination of t-SPL with dry etching avoids any additional chemical post-processing on PPA, such as cyclic infiltration of inorganic materials to convert PPA resists into etch-resistant inorganic hard masks after patterning [4].

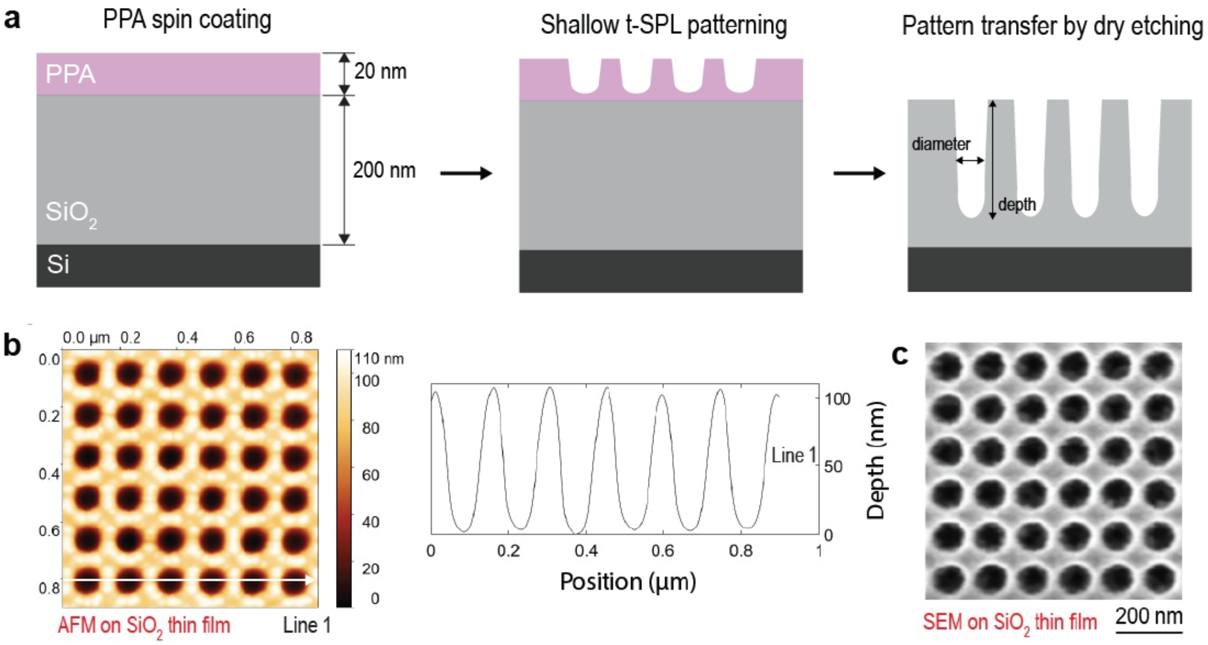

Figure S11: Depth amplification of binary nanopatterns. (a) Schematic of a shallow t-SPL for nanoholes, featuring nearly vertical walls and depth amplification into SiO_2. Shallow patterning with the tip apex results in steeper sidewalls. (b) AFM and (c) SEM images of nanohole arrays fabricated on thin film SiO_2 with 5.1 times depth amplification (CHF_3/SF_6 plasma with flow rate of 50/10 sccm at 950 W RF ICP power, 15 W RF bias power, and 5 mTorr pressure).

Section 8: Depth amplification of sinusoidal nanopatterns up to 10

About 10-fold depth amplification is achieved using CHF_3/SF_6 plasma with flow rate of 50/10 sccm at 950 W RF ICP power, 5 mTorr pressure, and 12 W RF bias power. In this condition for maximum depth amplification, the sinusoidal profiles are highly distorted (Fig. S12).

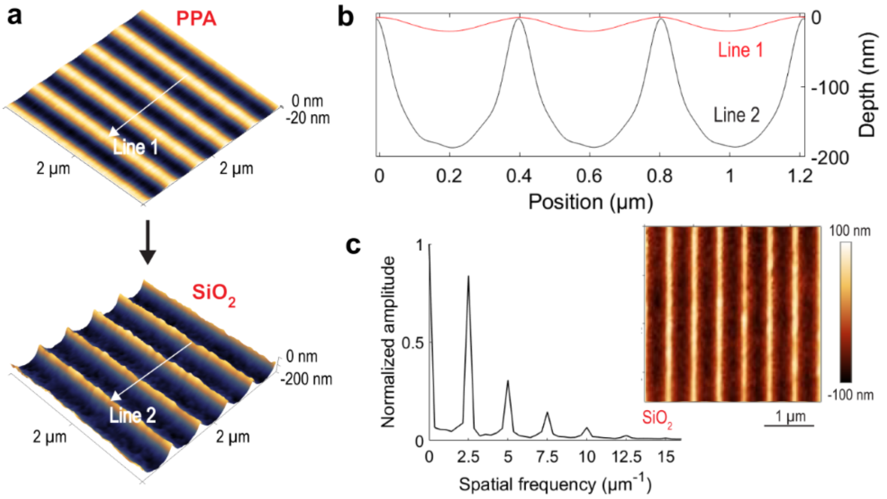

Figure S12: High depth amplification for sinusoidal nanopatterns. (a) AFM images and (b) amplitude comparison of sinusoidal nanopatterns before (Line 1) and after (Line 2) 9.5 times grayscale depth amplification. (c) Fourier transforms of the measured topographies on SiO_2.

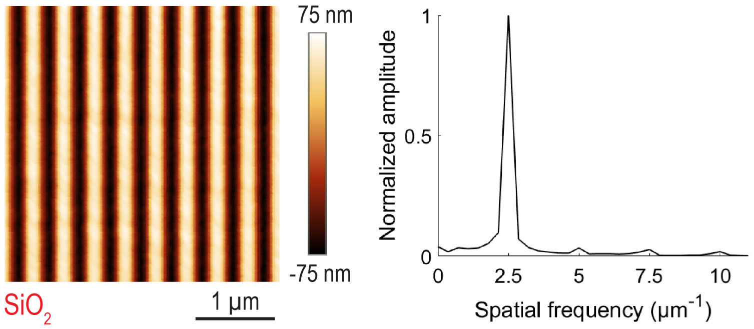

Figure S13: Depth amplification up to 5 times into SiO_2. Fourier transforms of the measured topographies after depth amplification of sinusoidal nanopatterns from 30 nm to 150 nm into SiO_2.

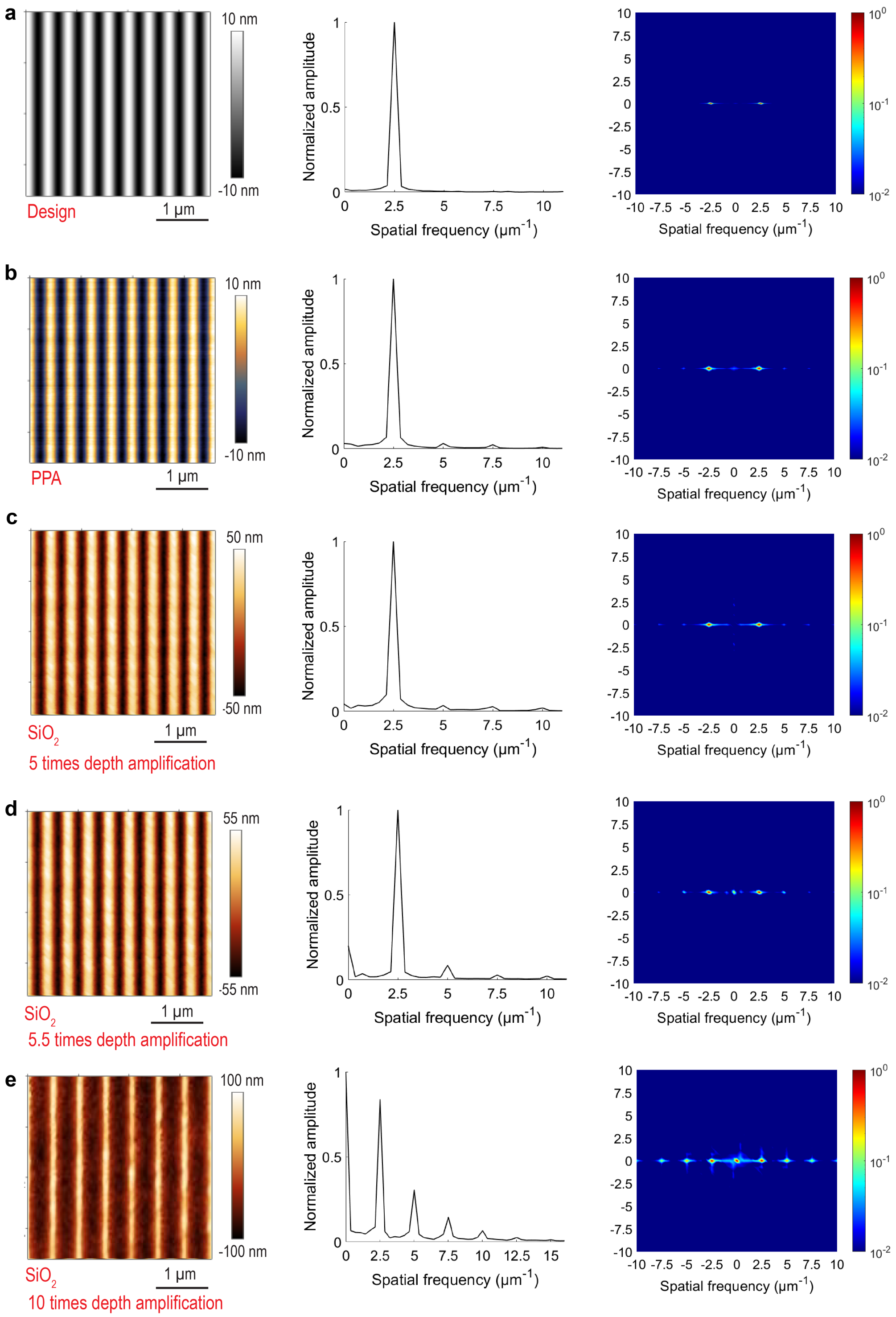

Figure S14: Topography characterization. Fourier transforms of (a) the design bitmap, (b) the measured topographies of the sinusoidal patterns after t-SPL on PPA, and (c) after 5 times, (d) after 5.5 times and (e) 10 times grayscale depth amplification on SiO_2.

Section 9: SEM images of dielectric grayscale nanosturcutres

After imaging grayscale nanostructures on thin films with AFM, we conducted SEM imaging on these dielectric films (Fig. S15). The charging effect makes imaging of dielectric thin films challenging, leading to difficulties in capturing images without signal saturation and distortion. Some drifts have also been observed when scanning through partly conducting and partly non-conducting regions across the electron beam. Additionally, the edges of structures appear brighter and exhibit altered contrast compared to the rest of the structure, a phenomenon that has been considered in explaining the observed minor distortion. In addition to Figure S15, images with high spatial and vertical resolution are presented in Figures 3 and S1.

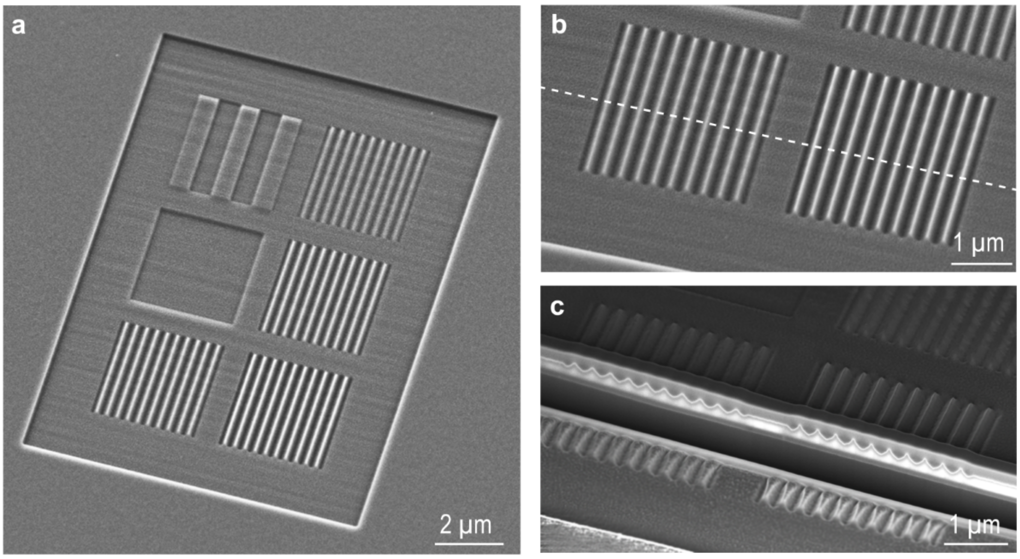

Figure S15: SEM images of structured SiO_2 thin films. SEM images of (a) grayscale nanopatterns and (b) sinusoidal patterns having 400 nm pitch with 100 nm and 150 nm peak-to-peak depths. Tilts are 30°. (c) 54° tilt corrected SEM image of sinusoidal patterns after ion-induced C protection layer deposition and 1 µm wide FIB milling along the dashed line in (b).

Section 10: Reactive ion etching and plasma parameters

The parameters of the dry etching recipes used for depth amplification while transferring the nanopatterns from PPA to SiO_2 are shown in Table S1. An ICP power of 950 W is used in the processes. Standard deviations are quantified by comparing the average peak-to-peak depths on PPA after t-SPL, and on SiO_2 after dry etching for a minimum 16 surface profiles measured by AFM. Different depths for sinusoidal and rectangular patterns are used for profile comparisons.

Table S1: Details of dry etch recipes and results for nanopattern transfer from PPA to SiO_2 and Si_3N_4 thin films.

Process #	Thin film dielectric	Gas composition	Flow (sccm)	Pressure (mTorr)	RF power (W)	Dry etching time (s)	Cooling time (min)	Depth amplification	Dielectric etch rate (nm/s)
1	SiO_2	CHF_3/SF_6	50/10	5	15	300	0	3.9±0.1	NA
2	SiO_2	CHF_3/SF_6	50/10	5	15	3x100	2	4.5±0.2	NA
3	SiO_2	CHF_3/SF_6	50/10	5	15	3x100	5	5.1±0.2	1.6
4	SiO_2	CHF_3/SF_6	50/10	5	15	3x100	10	5.1±0.1	1.6
5	SiO_2	CHF_3/SF_6	50/10	5	15	3x100	15	5.2±0.2	NA
6	SiO_2	CHF_3/SF_6	50/10	5	15	3x120	5	4.9±0.1	NA
7	SiO_2	CHF_3/SF_6	50/10	5	80	30	0	1.2±0.1	6.1
8	SiO_2	CHF_3/SF_6	50/10	5	40	100	0	1.8±0.1	3.8
9	SiO_2	CHF_3/SF_6	50/10	5	20	2x100	5	3.3±0.1	2.2
10	SiO_2	CHF_3/SF_6	50/10	5	18	2x100	5	4±0.1	1.9
11	SiO_2	CHF_3/SF_6	50/10	5	15	3x100	5	5.1±0.2	1.6
12	SiO_2	CHF_3/SF_6	50/10	5	14	4x100	5	5.5±0.2	1.4
13	SiO_2	CHF_3/SF_6	50/10	5	12	9x100	5	9.8±1	1
14	SiO_2	CHF_3/SF_6	50/10	15	15	100	0	3±0.1	2.3
15	SiO_2	CHF_3/SF_6	50/10	10	15	2x100	5	4.5±0.1	2
16	SiO_2	CHF_3/SF_6	50/10	5	15	3x100	5	5.1±0.2	1.6
17	SiO_2	CHF_3/SF_6	50/10	3.5	15	4x100	5	5.2±0.2	1.6
18	SiO_2	CHF_3/SF_6	50/10	3	15	4x100	5	4.8±0.2	1.4
19	SiO_2	CHF_3/SF_6	50/10	3.5	40	100	0	2±0.1	3.8
20	SiO_2	CHF_3/SF_6	50/10	3.5	20	2x100	5	3.9±0.1	2
21	SiO_2	CHF_3/SF_6	50/10	3.5	18	3x100	5	4.2±0.2	1.9
22	SiO_2	CHF_3/SF_6	50/10	3.5	15	4x100	5	5.2±0.2	1.6
23	SiO_2	CHF_3/SF_6	50/10	5	15	3x100	5	5.1±0.2	1.6
24	SiO_2	CHF_3/SF_6	50/15	5	15	2x100	5	2.7±0.2	2.4
25	SiO_2	CHF_3/SF_6	50/20	5	15	2x100	5	2±0.1	2.5
26	SiO_2	$CHF_3/SF_6/O_2$	50/10/5	5	15	3x100	5	2.6±0.1	1.9
27	SiO_2	$CHF_3/SF_6/O_2$	50/10/10	5	15	2x100	5	1.8±0.1	2
28	Si_3N_4	CHF_3/SF_6	50/10	5	40	100	0	1.7±0.1	4.5
29	Si_3N_4	CHF_3/SF_6	50/10	5	20	2x100	5	1.9±0.1	1.8
30	Si_3N_4	CHF_3/SF_6	50/10	5	18	2x100	5	2.1±0.1	1.4
31	Si_3N_4	CHF_3/SF_6	50/10	5	15	3x100	5	1.9±0.1	1
32	Si_3N_4	CHF_3/SF_6	50/10	5	14	3x100	5	1.5±0.1	0.7

Section 11: Replicated nanostructures by thermal NIL

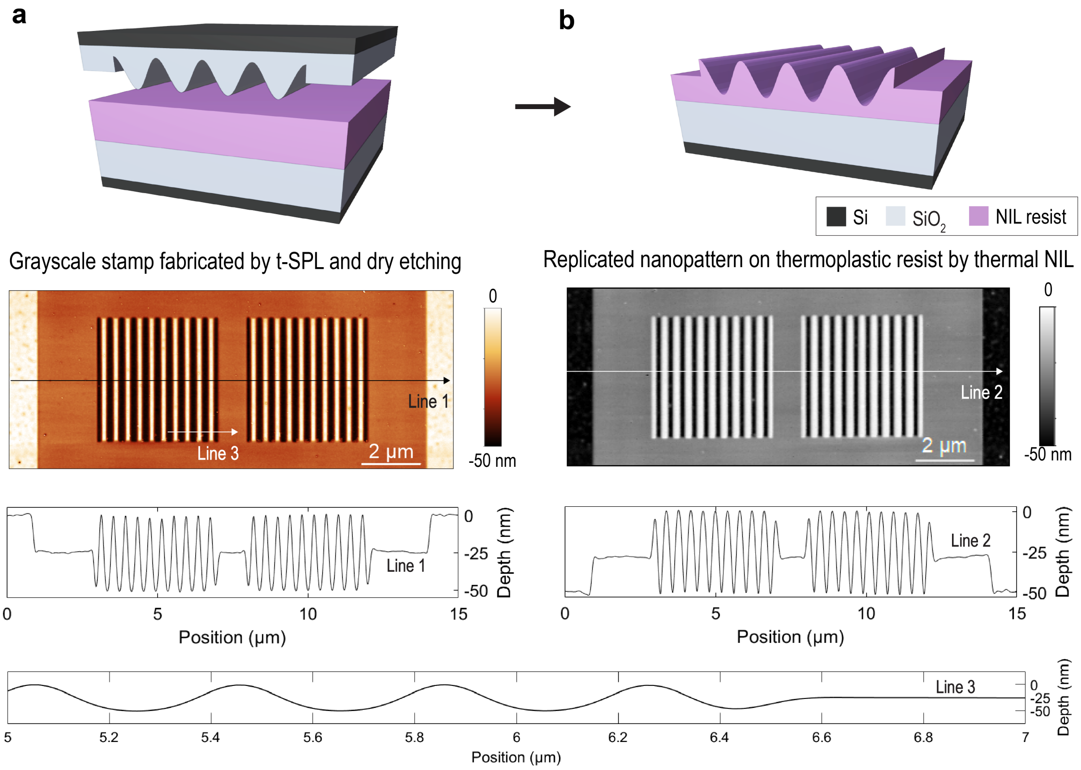

Figure S16: Thermal NIL. (a) AFM image and surface profile of grayscale stamp fabrication by combining t-SPL and dry etching. Line 3 shows 1:1 scale surface profile. (b) AFM image and surface profile of sinusoidal nanopatterns replicated on thermoplastic NIL resist mr-I 8010.

Section 12: Surface topography characterization for 2D materials

MoS_2 monolayers conformally follow sinusoidal SiO_2 surfaces. However, we still observe wrinkles in some parts of the flake. Figure S17 shows quantitative mechanical characterization to visualize this effect.

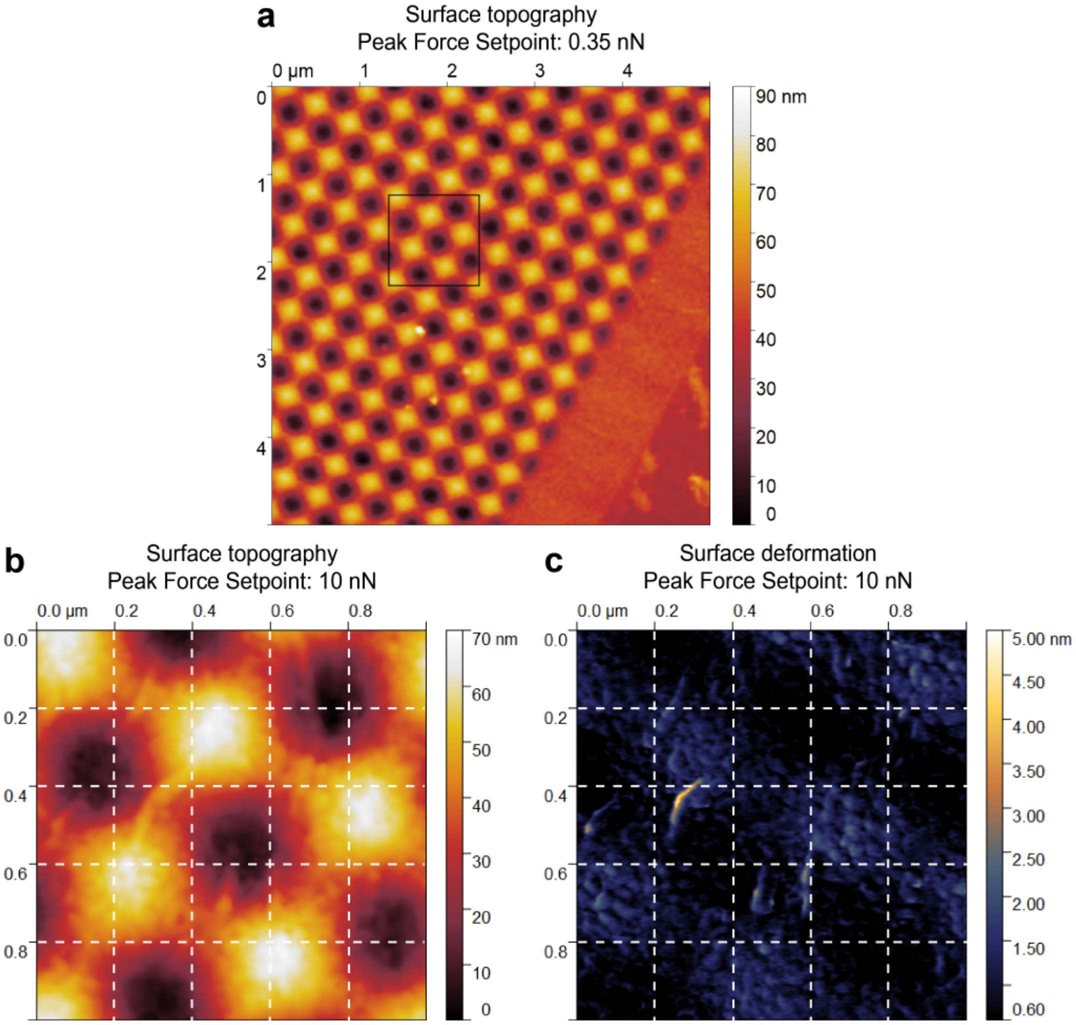

Figure S17: AFM on 2DMs. (a) Surface topography of sinusoidal surfaces with low peak forces. (b, c) Surface topography and deformation on 1 µm^2 area (black square in a), where wrinkles are present.

Conformal attachment of 2D material flake is further studied by TEM and energy dispersive X-ray (EDX) elemental mapping. TEM and EDX analyses serve as effective methods to assess the continuity of 2D flakes and their conformal adherence to the structured substrate.

A cross-sectional device lamella was prepared via FIB and SEM imaging. The selected area underwent carbon layer deposition to protect MoS_2 from surface damage. Perpendicular cutting of the lamella provided a view of the interface between MoS_2 and corrugated SiO_2.

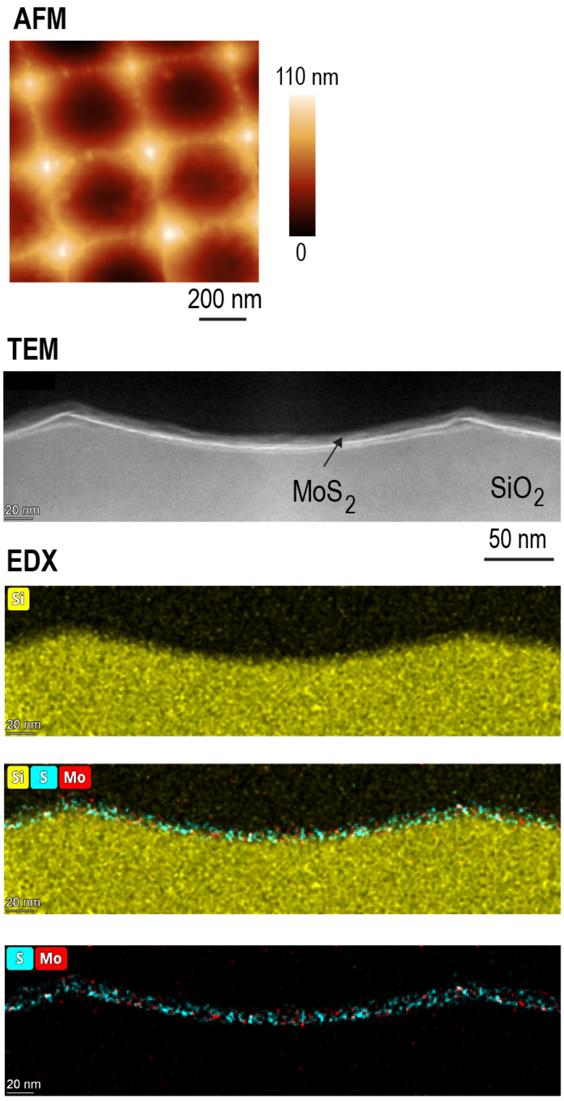

Figure S18: TEM and EDX. Cross-sectional TEM image of MoS_2 monolayer conformally following the structured SiO_2 and corresponding EDX elemental maps. TEM and EDX validation confirms that the 2DM flakes adhere intimately to the nanopatterned dielectric without any noticeable suspension or wrinkling.

Section 13: Strain characterization

Table S2: Raman micro-spectroscopy on monolayer MoS_2

Chip #	Pitch (nm)	Depth (nm)	Depth-to-pitch ratio	Flat MoS_2		Strained MoS_2		Shifts (cm^{-1})	
				$E^1_{2g}(cm^{-1})$	$A_{1g}(cm^{-1})$	$E^1_{2g}(cm^{-1})$	$A_{1g}(cm^{-1})$	E^1_{2g}	A_{1g}
1	500	60	0.12	385.4	404.5	384.8	403.8	-0.6	-0.7
1	500	100	0.20	385.4	404.5	384.7	403.5	-0.7	-1
1	500	100	0.20	385.4	404.5	384.3	403.5	-1.1	-1
2	500	80	0.16	385.9	404.8	385.2	403.8	-0.7	-1
2	500	120	0.24	385.9	404.8	384.3	404.2	-1.6	-0.6
3*	400	60	0.15	385.4	404.4	379.8/385	403.4	-5.6/0.4	-1
3	400	100	0.25	385.4	404.4	384.6	404	-0.8	-0.4
4	400	80	0.20	385.9	404.6	385	403.6	-0.9	-1
4	400	120	0.30	385.9	404.6	384.4	403.8	-1.5	-0.8
5	400	50	0.13	385.1	404.7	384.8	404.5	-0.3	-0.2
6	400	60	0.15	385.6	405	384.9	404.4	-0.7	-0.6
7	400	60	0.15	385.8	405.2	385	405.1	-0.8	-0.1
8*	300	60	0.2	386	404.4	378.1/384.8	404.1	-7.9/1.2	-0.3
8	300	100	0.33	386	404.4	384.4	404	-1.6	-0.4
9	300	80	0.27	385.6	404.6	384.5	404.5	-1.1	-0.1
10	300	70	0.23	386.1	405.3	385.2	404.9	-0.9	-0.3

* Two peaks are clearly observed.

E^1_{2g} Raman peak broadening is observed in strained MoS_2. However, in cases where clear peak splitting is not observed, only one Lorentzian function is fitted (Fig. S19). Lorentzian functions are fitted using Peakfit.m function in Matlab (version 9.9, https://terpconnect.umd.edu/ toh/spectrum/functions.html).

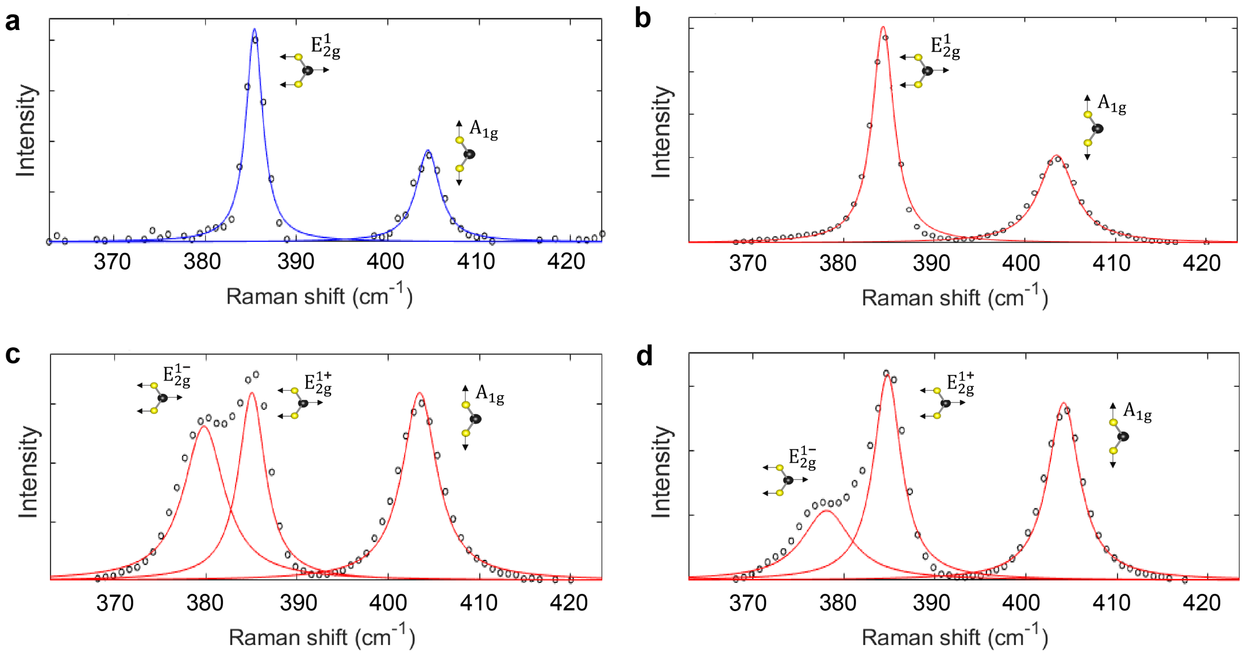

Figure S19: Raman micro-spectroscopy comparison of a MoS_2 monolayer with Lorentzian fits on (a) flat (chip #3), (b) sinusoidal waves with 500 nm pitch and 60 nm depths (chip #1), (c) sinusoidal waves with 400 nm pitch and 60 nm depths (chip #3), (d) sinusoidal waves with 300 nm pitch and 60 nm depths (chip #8). The insets show the schematic of the atomic vibration of the in-plane (E^1_{2g}) and out-of-plane (A_{1g}) modes.

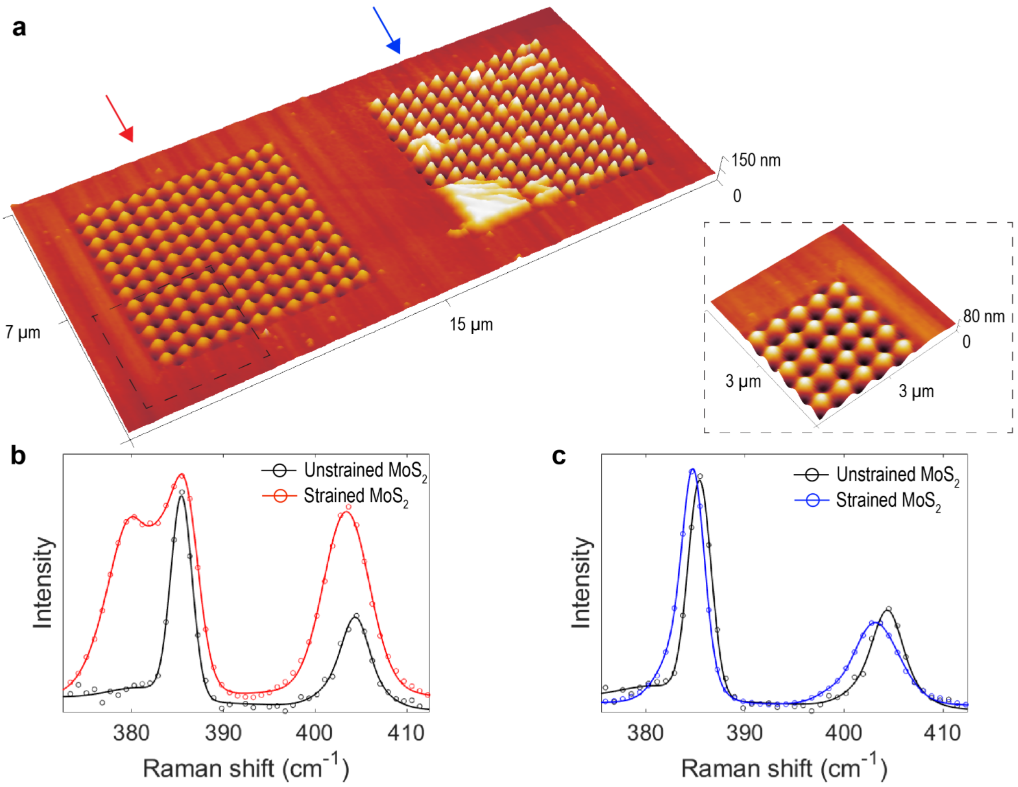

Figure S20: Strain characterization. (a) AFM image of chip #3 (Table 2) with 0.3 nN peak force setpoints. MoS_2 monolayer covers structured SiO_2 layer. Peak-to-peak depths are 60 nm in the left sinusoidal and 100 nm in the right sinusoidal. Both sinusoidal nanopatterns have a 400 nm pitch. (b, c) Raman micro-spectroscopy comparison of unstrained MoS_2 with strain MoS_2 monolayer on sinusoidal SiO_2 patterns. The intensities of Raman measurement obtained on the unstrained MoS_2 are amplified 40 times in b and 25 times in c. Lower Raman shift is observed on the area where MoS_2 is noticeably ripped.

References

1. Cheong, L. L. *et al.* Thermal probe maskless lithography for 27.5 nm half-pitch Si technology. *Nano letters* **13,** 4485–4491 (2013).

2. Rawlings, C. D. *et al.* Control of the interaction strength of photonic molecules by nanometer precise 3D fabrication. *Scientific Reports* **7,** 1–9 (2017).

3. Lisunova, Y., Spieser, M., Juttin, R., Holzner, F. & Brugger, J. High-aspect ratio nanopatterning via combined thermal scanning probe lithography and dry etching. *Microelectronic Engineering* **180,** 20–24 (2017).

4. Marneffe, J.-F. d. *et al.* Conversion of a patterned organic resist into a high performance inorganic hard mask for high resolution pattern transfer. *ACS nano* **12,** 11152–11160 (2018).
